# Identifying Multi-Dimensional Co-Clusters in Tensors Based on Hyperplane Detection in Singular Vector Spaces

**DOI:** 10.1371/journal.pone.0162293

**Published:** 2016-09-06

**Authors:** Hongya Zhao, Debby D. Wang, Long Chen, Xinyu Liu, Hong Yan

**Affiliations:** 1 Industrial Center, Shenzhen Polytechnic, Shenzhen, China; 2 Department of Electronic Engineering, City University of Hong Kong, Kowloon, Hong Kong; 3 Caritas Institute of Higher Education, New Territories, Hong Kong; Universita degli Studi del Piemonte Orientale Amedeo Avogadro, ITALY

## Abstract

Co-clustering, often called biclustering for two-dimensional data, has found many applications, such as gene expression data analysis and text mining. Nowadays, a variety of multi-dimensional arrays (tensors) frequently occur in data analysis tasks, and co-clustering techniques play a key role in dealing with such datasets. Co-clusters represent coherent patterns and exhibit important properties along all the modes. Development of robust co-clustering techniques is important for the detection and analysis of these patterns. In this paper, a co-clustering method based on hyperplane detection in singular vector spaces (***HDSVS***) is proposed. Specifically in this method, higher-order singular value decomposition (HOSVD) transforms a tensor into a core part and a singular vector matrix along each mode, whose row vectors can be clustered by a linear grouping algorithm (LGA). Meanwhile, hyperplanar patterns are extracted and successfully supported the identification of multi-dimensional co-clusters. To validate ***HDSVS***, a number of synthetic and biological tensors were adopted. The synthetic tensors attested a favorable performance of this algorithm on noisy or overlapped data. Experiments with gene expression data and lineage data of embryonic cells further verified the reliability of ***HDSVS*** to practical problems. Moreover, the detected co-clusters are well consistent with important genetic pathways and gene ontology annotations. Finally, a series of comparisons between ***HDSVS*** and state-of-the-art methods on synthetic tensors and a yeast gene expression tensor were implemented, verifying the robust and stable performance of our method.

## Introduction

Clustering analysis has become a fundamental tool in statistics, machine learning and signal processing [1]. A number of clustering algorithms have been developed, with the general idea of seeking groups among different objects in a full feature space. However, this process has several limitations, such as the adoption of a global-feature similarity among objects and the selection of a representative for each group. In contrast, many applications detect sub-matrices, which manifest coherent patterns among the rows or columns, in the object-feature matrix. For example, identification of genes that are co-expressed under certain conditions in microarray experiments and text mining of document groups that are characterized by word groups are two representatives of this type of study [2, 3]. To assist this sub-matrix analysis, a series of efficient approaches, such as sub-dimensional clustering, linear grouping and co-clustering, have been developed [2, 4–10].

Inspired by the concept of *direct clustering* [11], biclustering (co-clustering) plays an important role in the analysis of gene expression data [2]. This technique simultaneously clusters the rows (genes) and columns (experimental conditions) of the gene-condition matrix. Consequently, a subset of rows exhibiting significant coherence within a subset of columns in the matrix can be extracted. These coherent rows and columns are accordingly regarded as a bicluster, which corresponds to a specific coherent pattern. Commonly-studied biclusters in gene expression data present patterns with *constant value*, *coherent value*, and *coherent evolutions* [12]. Practically, biclustering is quite challenging, especially for large-scale data sets. Comprehensive reviews on this topic can be found in [12–17].

Cheng and Church developed an efficient node-detection algorithm (***CC***) to find valuable submatrices in yeast or human experssion data, based on mean squared residue scores [2]. Later, co-clustering in a document-word matrix was novelly transferred into a bipartite graph partitioning problem by Dhillon [18], and a spectral algorithm (***BSGP***) was proposed to give a reasonable partitioning solution. Bergmann *et al*. defined a transcription module in gene expression data by iteratively searching for co-clusters until a threshold was reached (***ISA***) [19]. ***ISA*** was also reported to perform well when applied to large-scale data. Subsequently, a simple binary reference model was provided for comparing and validating biclustering methods [20], and meanwhile a fast divide-and-conquer algorithm (***BiMax***: http://www.tik.ee.ethz.ch/sop/bimax) was proposed. As another classic co-clustering method, ***FABIA*** (*Factor Analysis for Bicluster Acquisition*) is a multiplicative model depending on linear dependencies between gene expression and experimental conditions [21].

Nowadays, multi-dimensional arrays (tensors), such as color images ([*row*, *column*, *color*]) [22, 23] and microarray data ([*gene*, *condition*, *time*]) [24, 25], frequently occur in clustering-related studies, demanding effective techniques that can deal with such data sets and identify useful co-clusters in them [26–30]. Banerjee *et al*. proposed a tensor co-clustering method by describing all the known relations between the different entity classes with a relation graph model [27]. Another method to detect co-clusters in tensors is based on multilinear decomposition with sparse latent factors [29]. In our work, the multi-dimensional co-clustering of a high-order tensor is accomplished by the conjunction of higher-order singular value decomposition (HOSVD) [23] and linear grouping algorithm (LGA) [5, 31]. Huang *et al*. has also employed HOSVD [23], toghther with the K-Means clustering, in their co-cluster method. However, the K-Means algorithm could only form clusters around “object” centers in the singular vector spaces, which is mainly related to constant biclusters. On the contrary, LGA could find linear structures (lines, planes and hyperplanes) in the singular vector spaces. These linear structures correspond to other types of co-clusters (constant-row/column, additive and mulitplicative co-clusters) in addition to constant ones in the original data. Firstly in our method, generalized from the SVD of a matrix, a truncated HOSVD is implemented on an *N*th-order tensor, resulting in a core tensor and a series of singular vector matrices along each mode. Secondly, LGA is subsequently applied to reveal the linear patterns embedded in singular vector matrices, with biclusters along each mode detected. Finally, through combining the detected linear structures of all the modes and defining a scoring function, we can successfully identify significant co-clusters in the tensor. To validate our method and compare it with existing ones, multiple synthetic and biological tensors are used, and the detected significant co-clusters are analyzed according to genetic pathways and gene ontology (***GO***) annotations [32] to attest the biological significance of these co-clusters [33].

## Methods

### Notations and Preliminaries

An *N*th-order (*N*-mode) tensor $A=\{a_{i_{1}i_{2}\ldots i_{N}},i_{k}=1,\ldots ,I_{k}\}$ can be defined as a multi-dimensional array, where *N* is the number of dimensions [23]. Here we adopt a boldface and Euler-script letter A to denote a tensor, with its entry notated as *a*_*i*_1_*i*_2_…*i*_*N*__. Accordingly, a column vector can be denoted using a boldface and lowercase letter, e.g. **a**, with its *i*th entry notated as *a*_*i*_; and a matrix is denoted by a boldface and uppercase letter, e.g. **A**, with its entry in the *i*th row and *j*th column denoted as *a*_*ij*_.

Further, the *i*th row and *j*th column of **A** can be notated as **a**_*i*:_ and **a**_:*j*_, respectively. Additionally, fibers and slices of A can be defined. For example, the column-, row- and tube-fibers of a 3-mode tensor $A\in R^{I_{1}\times I_{2}\times I_{3}}$ are denoted as **a**_:*jk*_, **a**_*i*:*k*_, and **a**_*ij*:_, respectively. The horizontal, lateral and frontal slices of this tensor are notated as **A**_*i*::_, **A**_:*j*:_, and **A**_:: *k*_, respectively.

The process of transforming a tensor into a 2D matrix is called unfolding or flattening. The mode-*n* unfolded matrix **A**_(*n*)_ of a tensor $A\in R^{I_{1}\times I_{2}\times \ldots \times I_{N}}$ is a matrix of size $I_{n}\times \left(\prod_{k\neq n}I_{k}\right)$, with its mode-*n* fibers reduced to the columns [34].

Unfolded matrices play an important role in the product of a tensor and a matrix. Similar to the product of two 2D matrices, the mode-*n* product of a tensor $A\in R^{I_{1}\times I_{2}\times \ldots \times I_{N}}$ with a matrix $U\in R^{J\times I_{n}}$ can be denoted as $A\times_{n}U$, which is a tensor (size *I*_1_ × … × *I*_*n*−1_ × *J* × *I*_*n*+1_ × … × *I*_*N*_) composed of the following entries,

$$\begin{array}{c} {\left(A\times_{n}U\right)}_{i_{1}\ldots i_{n-1}ji_{n+1}\ldots i_{N}}=\sum_{i_{n}=1}^{I_{n}}a_{i_{1}\ldots i_{n-1}i_{n}i_{n+1}\ldots i_{N}}u_{ji_{n}} \end{array}$$ (1)

This can also be described by a matrix product in Eq (2), using the unfolded matrices.

$$\begin{array}{c} y=A\times_{n}U\Leftrightarrow Y_{\left(n\right)}={\mathrm{UA}}_{\left(n\right)} \end{array}$$ (2)

### Biclustering in Matrices

#### Biclusters in Matrices

In a data matrix **A**, a bicluster is a sub-matrix of **A** and represents a coherent pattern [12]. Specifically, we notate a bicluster as **A**_**IJ**_, where **I** = {*i*_1_, *i*_2_, …, *i*_*s*_} stands for a subset of rows and **J** = {*j*_1_, *j*_2_, …, *j*_*t*_} is a subset of columns. Based on **A**_**IJ**_, we can further define several types of generally-discussed biclusters:

1. constant biclusters, i.e. {*a*_*ij*_ = *μ* ∣ *i* ∈ **I**, *j* ∈ **J**};
2. constant-row or constant-column biclusters, i.e. $\left\{a_{iJ}^{T}=\mu_{i}1_{J}|i\in I\right\}$ or {**a**_**I***j*_ = *μ*_*j*_ **1**_**I**_ ∣ *j* ∈ **J**} where **1**_**J**_ or **1**_**I**_ is a column vector of ones;
3. additive-row or additive-column biclusters, i.e. $\left\{a_{iJ}^{T}=\mu_{i}1_{J}+a_{kJ}^{T}|i,k\in I\right\}$ or {**a**_**I***j*_ = *μ*_*j*_ **1**_**I**_ + **a**_**I***k*_ ∣ *j*, *k* ∈ **J**};
4. multiplicative-row or multiplicative-column biclusters, i.e. $\left\{a_{iJ}^{T}=\mu_{i}a_{kJ}^{T}|i,k\in I\right\}$ or {**a**_**I***j*_ = *μ*_*j*_ **a**_**I***k*_ ∣ *j*, *k* ∈ **J**}.

Let us consider the analysis of gene expression data ([*gene*, *experimental condition*]) as an example. Our aim is to identify gene groups with similar behaviors or functions, such as a group of genes that are highly correlated under a group of experimental conditions [2, 12, 35]. In this regard, a constant bicluster means that a group of genes have the same expression level under a group of conditions, thus exhibiting certain kinds of homogeneity [35]. Similarly, constant-row, constant-colonm, addtive and multiplicative biclusters can also reveal genes with related behaviours or functions, which can be coordinately investigated and targeted in gene regulation [6, 12]. Specifically, a constant-row bicluster means that each gene in a group has the same expression level under all conditions in a group, but different genes may have different expression levels. A constant-column bicluster means that all genes in a group have the same expression level under each condition in a group, but a gene may have different expression levels for different conditions. In an additive bicluster, expression levels of all genes in a group under one condition is higher or lower by a constant than those under another condition. In a multiplicative bicluster, expression levels of all genes in a group under one condition is a multiple of those under another condition.

Most biclustering techniques permutate the original matrix and optimize a scoring function. Commonly-used scoring functions include the sum of squares [11] in Eq (3) and the mean squared residue score [2] in Eq (4),

$$\begin{array}{c} SSQ=\sum_{i\in I,j\in J}{\left(a_{ij}-{\bar{A}}_{\mathrm{IJ}}\right)}^{2} \end{array}$$ (3)

$$\begin{array}{c} H\left(I,J\right)=\frac{1}{\left|I||J\right|}\sum_{i\in I,j\in J}{\left(a_{ij}-{\bar{a}}_{iJ}-{\bar{a}}_{Ij}+{\bar{A}}_{\mathrm{IJ}}\right)}^{2} \end{array}$$ (4)

where ${\bar{A}}_{\mathrm{IJ}}$ is the mean of sub-matrix **A**_**IJ**_, and ${\bar{a}}_{iJ}$ and ${\bar{a}}_{Ij}$ are the means of row **a**_*i***J**_ and column **a**_**I***j*_, respectively. **A**_**IJ**_ is defined as a *δ*-bicluster if *H*(**I**, **J**) ≤ *δ*, where *δ*(*δ* > 0) is a pre-specified residue score threshold value.

Unfortunately, *H*(**I**, **J**) can be used only to detect biclusters of types (a), (b) and (c), but not type (d). Therefore, a more general scoring function was proposed [36] for a thorough bicluster-search. This function *S*(**I**, **J**), as expressed in Eq (5), is derived from Pearson’s correlation:

$$\begin{array}{c} S\left(I,J\right)={\text{min}}_{i\in I,j\in J}\left(S_{Ij},S_{iJ}\right) \end{array}$$ (5)

where $S_{Ij}=1-\frac{1}{|J|-1}\sum_{j\neq k\in J}\left|\rho \left(a_{Ij},a_{Ik}\right)\right|$ and $S_{iJ}=1-\frac{1}{|I|-1}\sum_{i\neq k\in I}\left|\rho \left(a_{iJ},a_{kJ}\right)\right|$. $\rho \left(x,y\right)=\frac{\sum \left(x-\bar{x}\right)\left(y-\bar{y}\right)}{\sqrt{\sum {\left(x-\bar{x}\right)}^{2}}\sqrt{\sum {\left(y-\bar{y}\right)}^{2}}}$ is Pearson’s correlation between two vectors **x** and **y**. Normally, a lower *S*(**I**, **J**) value represents a stronger coherence among the involved rows or columns [36]. Similarly as above, we can define a *δ*-bicluster if *S*(**I**, **J**) ≤ *δ*, where *δ* > 0.

#### Biclustering Based on Singular Value Decomposition (SVD) and Clustering in Singular Vector Matrices

In biclustering analysis, SVD-based methods play an important role and have been broadly applied to detect significant biclusters. Representative methods include sparse SVD (SSVD), regularized SVD (RSVD), robust regularized SVD (RobRSVD), nonnegative matrix factorization (NMF) and nonsmooth-NMF (nsNMF) [36–39].

Let **A** be a matrix of size *N* × *M*, and its SVD can be defined as follows,

$$\begin{array}{c} A=U\Sigma V^{T}=\sum_{i=1}^{r}\sigma_{i}u_{i}v_{i}^{T} \end{array}$$ (6)

where *r* is the rank of **A**, **U** = [**u**_1_ **u**_2_ … **u**_*r*_] is an *N* × *r* matrix of left orthonormal singular vectors, **V** = [**v**_1_ **v**_2_ … **v**_*r*_] is an *M* × *r* matrix of right orthonormal singular vectors, and **Σ** = *diag*(*σ*_1_ *σ*_2_ … *σ*_*r*_) is an *r* × *r* diagonal matrix with positive singular values (*σ*_1_ ≥ *σ*_2_ ≥ … ≥ *σ*_*r*_).

In Eq (6), $\sigma_{i}u_{i}v_{i}^{T}$ is a rank-one matrix that is called an SVD layer. Normally, the SVD layers corresponding to large *σ*_*k*_ values are regarded as effective signals, while the rest are considered as noise [36]. Based on effective SVD layers, a rank-*l* (*l* < *r*) approximation of **A** can be derived by minimizing the squared Frobenius norm:

$$\begin{array}{c} \begin{array}{cc} A\approx A^{\left(l\right)} & =\sum_{i=1}^{l}\sigma_{i}u_{i}v_{i}^{T}\\ & =\text{arg}\;{\text{min}}_{rank(A^{*})=l}{∥A-A^{*}∥}_{F}^{2}\\ & =\text{arg}\;{\text{min}}_{rank(A^{*})=l}\text{tr}\left\{\left(A-A^{*}\right){\left(A-A^{*}\right)}^{T}\right\} \end{array} \end{array}$$ (7)

In order to develop SVD-based techniques for co-cluster analysis of high-order tensors, let us first study the properties of biclusters in 2D singular vector spaces. For simplicity, merely coherent patterns along columns are considered here.

**Proposition 1:** If **A**_*c*_ is a bicluster with size *n* × *d*, then *rank*(**A**_*c*_) ≤ 2.

**Proof:** First, we can rewrite **A**_*c*_ as follows,

$$\begin{array}{c} A_{c}=\left[a_{:1}^{c}\;a_{:2}^{c}\;\ldots \;a_{:d}^{c}\right] \end{array}$$ (8)

If **A**_*c*_ corresponds to a constant or constant-column bicluster, then *rank*(**A**_*c*_) ≤ 1. Otherwise, if **A**_*c*_ corresponds to a multiplicative-column bicluster, then **A**_*c*_ can be formulated as Eq (9),

$$\begin{array}{c} A_{c}=\left[a_{:1}^{c}\;k_{2}a_{:1}^{c}\;\ldots \;k_{d}a_{:1}^{c}\right] \end{array}$$ (9)

As well, here the rank of **A**_*c*_ is no more than one (*rank*(**A**_*c*_) ≤ 1), since $k_{2}a_{:1}^{c},\ldots ,k_{d}a_{:1}^{c}$ are linearly dependent on $a_{:1}^{c}$.

At last, if **A**_*c*_ corresponds to an additive bicluster, then the following equation can be derived,

$$\begin{array}{c} A_{c}=\left[a_{:1}^{c}\;a_{:1}^{c}+b_{2}1_{n}\;\ldots \;a_{:1}^{c}+b_{d}1_{n}\right] \end{array}$$ (10)

Here, at most two vectors, such as $a_{:1}^{c}$ and $a_{:1}^{c}+b_{2}1_{n}$, are linearly independent. Therefore, *rank*(**A**_*c*_) ≤ 2.

**Proposition 2:** Assume **A**_*c*_ with size *s* × *t* is a bicluster. If $A_{c}=U_{c}\Sigma_{c}V_{c}^{T}$, where **U**_*c*_ and **V**_*c*_ are the left and right singular vector matrices respectively of **A**_*c*_ and $\Sigma_{c}=\text{diag}\left(\sigma_{1}^{c},\sigma_{2}^{c},\ldots ,\sigma_{s}^{c}\right)$ contains the singular values of **A**_*c*_ with $\sigma_{1}^{c}\geq \sigma_{2}^{c}\geq \ldots \geq \sigma_{s}^{c}$, then each column of **A**_*c*_ can be represented as a linear combination of first two columns, $u_{1}^{c}$ and $u_{2}^{c}$, of **U**_*c*_, and each row of **A**_*c*_ can be represented as a linear combination of first two columns, $v_{1}^{c}$ and $v_{2}^{c}$, of **V**_*c*_.

**Proof:** According to **Proposition 1**, the rank of **A**_*c*_ is at most 2. Because $\sigma_{m}^{c}=0$ for *m* > 2, $A_{c}=U_{c}\Sigma_{c}V_{c}^{T}$ can be rewritten as

$$\begin{array}{c} \begin{array}{cc} A_{c} & =U_{c}\Sigma_{c}V_{c}^{T}\\ & =\left[\begin{array}{cc} u_{1}^{c} & u_{2}^{c} \end{array}\right]\left[\begin{array}{cc} \sigma_{1}^{c} & 0\\ 0 & \sigma_{2}^{c} \end{array}\right]\left[\begin{array}{c} {\left(v_{1}^{c}\right)}^{T}\\ {\left(v_{2}^{c}\right)}^{T} \end{array}\right]\\ & =\left[\begin{array}{cc} u_{1}^{c} & u_{2}^{c} \end{array}\right]\left[\begin{array}{cc} \sigma_{1}^{c} & 0\\ 0 & \sigma_{2}^{c} \end{array}\right]\left[\begin{array}{cccc} v_{11}^{c} & v_{12}^{c} & \cdots & v_{1d}^{c}\\ v_{21}^{c} & v_{22}^{c} & \cdots & v_{2d}^{c} \end{array}\right]\\ & =\left[\begin{array}{cccc} \sigma_{1}^{c}v_{11}^{c}u_{1}^{c}+\sigma_{2}^{c}v_{21}^{c}u_{2}^{c} & \sigma_{1}^{c}v_{12}^{c}u_{1}^{c}+\sigma_{2}^{c}v_{22}^{c}u_{2}^{c} & \cdots & \sigma_{1}^{c}v_{1d}^{c}u_{1}^{c}+\sigma_{2}^{c}v_{2d}^{c}u_{2}^{c} \end{array}\right] \end{array} \end{array}$$ (11)

Let $\alpha_{j1}=\sigma_{1}^{c}v_{1j}^{c}$ and $\alpha_{j2}=\sigma_{2}^{c}v_{2j}^{c}$, then the *j*-th column of **A**_*c*_ is

$$\begin{array}{c} a_{j}^{c}=\alpha_{j1}u_{1}^{c}+\alpha_{j2}u_{2}^{c} \end{array}$$ (12)

That is,

$$\begin{array}{c} \left\{\begin{array}{c} \alpha_{j1}u_{11}^{c}+\alpha_{j2}u_{12}^{c}=a_{1j}^{c}\\ \alpha_{j1}u_{21}^{c}+\alpha_{j2}u_{22}^{c}=a_{2j}^{c}\\ \vdots \\ \alpha_{j1}u_{s1}^{c}+\alpha_{j2}u_{s2}^{c}=a_{sj}^{c} \end{array}\right. \end{array}$$ (13)

Thus, each column of **A**_*c*_ can be represented as a linear combination of first two columns, $u_{1}^{c}$ and $u_{2}^{c}$, of **U**_*c*_. Geometrically, Eqs (12) and (13) mean that points $\left(u_{m1}^{c},u_{m2}^{c}\right)\left(m=1,2,\ldots ,s\right)$ are distributed on a line.

Similarly, we can obtain

$$\begin{array}{c} a_{i}^{c}=\beta_{i1}v_{1}^{c}+\beta_{i2}v_{2}^{c} \end{array}$$ (14)

and

$$\begin{array}{c} \left\{\begin{array}{c} \beta_{i1}v_{11}^{c}+\beta_{i2}v_{12}^{c}=a_{i1}^{c}\\ \beta_{i1}v_{21}^{c}+\beta_{i2}v_{22}^{c}=a_{i2}^{c}\\ \vdots \\ \beta_{i1}v_{t1}^{c}+\beta_{i2}v_{t2}^{c}=a_{it}^{c} \end{array}\right. \end{array}$$ (15)

Thus, each row of **A**_*c*_ can be represented as a linear combination of first two columns, $v_{1}^{c}$ and $v_{2}^{c}$, of **V**_*c*_. Geometrically, Eqs (14) and (15) mean that points $\left(v_{m1}^{c},v_{m2}^{c}\right)\left(m=1,2,\ldots ,t\right)$ are distributed on a line.

**Proposition 3:** Assume **A**_*c*1_ and **A**_*c*2_ with sizes *s*_1_ × *t* and *s*_2_ × *t* are two different biclusters, where *s*_1_+*s*_2_ = *s*. Let $A_{c}=[\begin{array}{c} A_{c1}\\ A_{c2} \end{array}]$. If $A_{c}=U_{c}\Sigma_{c}V_{c}^{T}$, where **U**_*c*_ and **V**_*c*_ are the left and right singular vectors matrices respectively of **A**_*c*_ and $\Sigma_{c}=\text{diag}\left(\sigma_{1}^{c},\sigma_{2}^{c},\ldots ,\sigma_{s}^{c}\right)$ contains the singular values of **A**_*c*_ with $\sigma_{1}^{c}\geq \sigma_{2}^{c}\geq \ldots \geq \sigma_{s}^{c}$, then each column of **A**_*c*1_ can be represented as a linear combination of *s*_1_ rows of first four columns, $u_{1}^{c1}$, $u_{2}^{c1}$, $u_{3}^{c1}$, $u_{4}^{c1}$, of **U**_*c*_, each column of *A*_*c*2_ can be represented as another linear combination of rows *s*_1_ + 1 to *s*_1_ + *s*_2_ of first four columns, $u_{1}^{c2}$, $u_{2}^{c2}$, $u_{3}^{c2}$, $u_{4}^{c2}$, of **U**_*c*_, and each row of **A**_*c*_ can be represented as a linear combination of first four columns, $v_{1}^{c}$, $v_{2}^{c}$, $v_{3}^{c}$, $v_{4}^{c}$, of **V**_*c*_.

**Proof:** According to **Proposition 1**, the rank of **A**_*c*1_ is at most 2, and so is the rank of **A**_*c*2_. Thus, *rank*(**A**_*c*_) ≤ 4. Let $U_{c}=[\begin{array}{c} U_{c1}\\ U_{c2} \end{array}]$, where **U**_*c*1_ represents the first *s*_1_ rows and **U**_*c*2_ the remaining *s*_2_ rows of **U**_*c*_. Because $\sigma_{m}^{c}=0$ for *m* > 4, $A_{c}=U_{c}\Sigma_{c}V_{c}^{T}$ can be rewritten as

$$\begin{array}{c} \begin{array}{c} \begin{array}{c} A_{c}=\left[\begin{array}{c} A_{c1}\\ A_{c2} \end{array}\right]\\ =\left[\begin{array}{c} \left[\begin{array}{cccc} u_{1}^{c1} & u_{2}^{c1} & u_{3}^{c1} & u_{4}^{c1} \end{array}\right]\\ \left[\begin{array}{cccc} u_{1}^{c2} & u_{2}^{c2} & u_{3}^{c2} & u_{4}^{c2} \end{array}\right] \end{array}\right]\left[\begin{array}{ccccc} \sigma_{1}^{c} & & & & \\ & \sigma_{2}^{c} & & & \\ & & \sigma_{3}^{c} & & \\ & & & \sigma_{4}^{c} & \end{array}\right]\left[\begin{array}{c} {\left(v_{1}^{c}\right)}^{T}\\ {\left(v_{2}^{c}\right)}^{T}\\ {\left(v_{3}^{c}\right)}^{T}\\ {\left(v_{4}^{c}\right)}^{T} \end{array}\right] \end{array} \end{array} \end{array}$$ (16)

where $u_{m}^{c1}$ (*m* = 1, 2, 3, 4) are the first four columns of **U**_*c*1_ and $u_{m}^{c2}$ (*m* = 1, 2, 3, 4) are the first four columns of **U**_*c*2_. Similar to the proof for **Proposition 2**, we can obtain

$$\begin{array}{c} a_{j}^{c1}=a_{j1}^{c1}u_{1}^{c1}+a_{j2}^{c1}u_{2}^{c1}+a_{j3}^{c1}u_{3}^{c1}+a_{j4}^{c1}u_{4}^{c1} \end{array}$$ (17)

$$\begin{array}{c} a_{j}^{c2}=a_{j1}^{c2}u_{1}^{c2}+a_{j2}^{c2}u_{2}^{c2}+a_{j3}^{c2}u_{3}^{c2}+a_{j4}^{c2}u_{4}^{c2} \end{array}$$ (18)

where $a_{j}^{c1}$ represents the *j*-th column of **A**_*c*1_ or the first *s*_1_ elements in the *j*-th column of **A**_*c*_, and $a_{j}^{c2}$ represents the *j*-th column of **A**_*c*2_ or the remaining *s*_2_ elements in the *j*-th column of **A**_*c*_. Geometrically, Eq (17) means that points $\left(u_{m1}^{c},u_{m2}^{c},u_{m3}^{c},u_{m4}^{c}\right)$ (*m* = 1, 2, 3, …, *s*_1_) are distributed on a hyperplane, and that points $\left(u_{m1}^{c},u_{m2}^{c},u_{m3}^{c},u_{m4}^{c}\right)$ (*m* = *s*_1_ + 1, *s*_1_ + 2, *s*_1_ + 3, …, *s*_1_ + *s*_2_) are distributed on another hyperplane. Similarly, we can show that the points $\left(v_{m1}^{c},v_{m2}^{c},v_{m3}^{c},v_{m4}^{c}\right)$ (*m* = 1, 2, …, *t*) are also distributed on a hyperplane.

In practical applications, biclusters are embedded in a large matrix with irrelevant elements. Additionally, the biclusters themselves can have noise. Three examples are shown below:

$$\begin{array}{c} \begin{array}{ccccccc} XXXXXXXXXX & & & XXXXXXXXXX & & & XXXXXXXXXX\\ X\mathrm{AA}XXXAXXX & & & XAXAXXAXXX & & & XAXXXAXXXA\\ XXXXXXXXXX & & & XAXAXXAXXX & & & XXXXXXXXXX\\ XXXXXXXXXX & & & XXXXXXXXXX & & & XAXXXAXXXA\\ X\mathrm{AA}XXXAXXX & & & XAXAXXAXXX & & & XAXXXAXXXA\\ XXXXXXXXXX & & & XXXXXXXXXX & & & XXXXXXXXXX\\ XXXXXXXXXX & & & XBXBXXBXXX & & & XXBXBBXXXX\\ X\mathrm{AA}XXXAXXX & & & XXXXXXXXXX & & & XXBXBBXXXX\\ XXXXXXXXXX & & & XBXBXXBXXX & & & XXXXXXXXXX\\ XXXXXXXXXX & & & XBXBXXBXXX & & & XXBXBBXXXX\\ \text{Matrix}1 & & & \text{Matrix}2 & & & \text{Matrix}3 \end{array} \end{array}$$

In these matrices, “*X*” represents entries of irrelevant elements, and “**A**” and “**B**” represent the entries of two biclusters **A** and **B** respectively. The actual values at the location marked by “*X*” can be different, and they are background noise. The values at the locations marked by “**A**” and “**B**” should form bicluster patterns as described in Section “Biclusters in Matrices”.

Due to noise, the rank of Matrix 1 can be greater than 2. We can remove small singular values using a method to be discussed in Section “HOSVD”. Assume that we still retain 2 singular values, then Eqs (12) to (15) are only approximations for rows 2, 5 and 8. Remember that our task in biclustering is to find these row indices (and column indices 2, 3 and 7). That is, we do not know beforehand rows 2, 5 and 8 contain a bicluster and we need to identify them. Assume that we take SVD of Matrix 1, then from the left singular vector matrix **U**, points (*u*_*m*1_, *u*_*m*2_) (*m* = 2, 5, 8) should form a line approximately according to Eqs (12) and (13), while points (*u*_*m*1_, *u*_*m*2_) (*m* ≠ 2, 5, 8) will be distributed randomly and do not satisfy these equations. Our task now is to detect the line from all points (*u*_*m*1_, *u*_*m*2_) (1 ≤ *m* ≤ 10). Once the line is detected, we can then determine which points are on the line. The row indices of these points correspond to the locations of the bicluster. Similarly, we can identify relevant columns of the bicluster by detecting lines using the right singular vector matrix **V**. To identify biclusters in Matrices 2 and 3, we need to find hyperplanes in singular vector spaces. The lines and hyperplanes can be detected using the linear grouping algorithm (LGA) to be discussed in Section “Linear Grouping Algorithm (LGA)”.

### Identification of Co-clusters in High-order Tensors

#### Co-clusters in High-order Tensors

The hyperplane model in singular vector spaces can be extended to the analysis of higher-order tensor data. For example, a co-cluster (represented by a sub-tensor $A_{IJK}\in R^{I\times J\times K}$) in a 3-mode (3D) tensor $A_{I_{1}I_{2}I_{3}}\in R^{I_{1}\times I_{2}\times I_{3}}$), where {**I** ∈ **I**_1_, **J** ∈ **I**_2_, **K** ∈ **I**_3_}, can be similarly defined as in the 2D case. This definition involves the pre-defined fibers and slices in a 3D tensor. Now we use constant co-clusters as examples,

1. $A_{IJK}\in R^{I\times J\times K}$ corresponds to a constant co-cluster if {*a*_*ijk*_ = *μ* ∣ *i* ∈ **I**, *j* ∈ **J**, *k* ∈ **K**};
2. a constant-column-fiber co-cluster can be expressed as {**a**_**I***jk*_ = *μ*_*jk*_ **1**_**I**_ ∣ *j* ∈ **J**, *k* ∈ **K**};
3. a constant-horizontal-slice co-cluster can be defined as {**A**_*i***JK**_ = *μ*_*i*_ **1**_**JK**_ ∣ *i* ∈ **I**};

Here **1**_**I**_ or **1**_**JK**_ is a vector or matrix of ones. Accordingly, additive and multiplicative co-clusters can be also defined.

To evaluate the significance of co-clusters in high-order tensors, a scoring function generalized from that for matrices in Eq (5) can be used. Let *S*_**I**_1_…*i*_*n*_…**I**_*N*__ represent the average of *S*_**I**_1_*i*_*n*__, …, *S*_**I**_*n*−1_*i*_*n*__, *S*_*i*_*n*_**I**_*n*+1__, …, *S*_*i*_*n*_**I**_*N*__, then we can define the scoring function as

$$\begin{array}{c} \begin{array}{c} S(I_{1},I_{2},\ldots ,I_{N})\\ =min_{i_{1}\in I_{1},i_{2}\in I_{2},\ldots ,i_{N}\in I_{N}}\left(S_{i_{1}I_{2}\ldots I_{N}},S_{I_{1}i_{2}\ldots I_{N}},S_{I_{1}I_{2}\ldots i_{N}}\right) \end{array} \end{array}$$ (19)

Similarly, a lower score indicates a higher significance. A *δ*-co-cluster can be further defined if *S*(**I**_1_, **I**_2_, …, **I**_*N*_) ≤ *δ* (*δ* > 0). In particular, we name such a co-cluster in a 3D tensor as a *δ*-tricluster.

#### HOSVD

Similar to SVD, HOSVD that decomposes a tensor into a core tensor and a singular vector matrix along each mode is employed in our method, to extract co-clusters in high-order tensors [23, 40, 41].

The HOSVD of an *N*-mode tensor $A\in R^{I_{1}\times I_{2}\times \ldots \times I_{N}}$ can be expressed as follows,

$$\begin{array}{c} A=T\times_{1}U^{\left(1\right)}\ldots \times_{N}U^{\left(N\right)} \end{array}$$ (20)

where $U^{\left(n\right)}\in R^{I_{n}\times r_{n}}\;\left(n=1,\ldots ,N\right)$ is a factor (singular vector) matrix, and $T\in R^{r_{1}\times r_{2}\times \ldots \times r_{N}}$ is the core tensor. The SVD of a matrix **A** in Eq (6) can be formulated in a similar format with $T=\Sigma \in R^{I_{1}\times I_{2}}$, as a special case of Eq (20).

$$\begin{array}{c} A=U\Sigma V^{T}=\Sigma \times_{1}U\times_{2}V \end{array}$$ (21)

On the other hand, HOSVD can be expressed as a matrix format, using the unfolded matrices along each mode,

$$\begin{array}{c} A_{\left(n\right)}=U^{\left(n\right)}\Sigma^{\left(n\right)}{\left(V^{\left(n\right)}\right)}^{T},\;n=1,\ldots ,N \end{array}$$ (22)

That is, we obtain a matrix **A**_(*n*)_ of size *I*_*n*_ × *I*_1_*I*_2_⋯*I*_*n*−1_*I*_*n*+1_⋯*I*_*N*_ for mode *n*, and **U**^(*n*)^ is the left singular vector matrix of **A**_(*n*)_. When the tensor A is unfolded to a matrix **A**_(*n*)_, a co-cluster in A will be unfolded to a bicluster in **A**_(*n*)_. From **U**^(*n*)^, we can find the row indices of **A**_(*n*)_ that contain a bicluster, according to **Propositions 1** to **3**. These row indices correspond to the locations in the tensor along mode *n*. By combining the row indices of biclusters in all unfolded matrices **A**_(*n*)_ (*n* = 1, …, *N*), we can then find the co-cluster in an *N*-dimensional space or *N*-mode tensor A. Now the major task is to find hyperplanes for each singular vector matrix **U**^(*n*)^. That is, the problem of detecting co-clusters in a multi-dimensional space has been effectively converted to the detection of biclusters in singular vector spaces.

**Fig 1:**
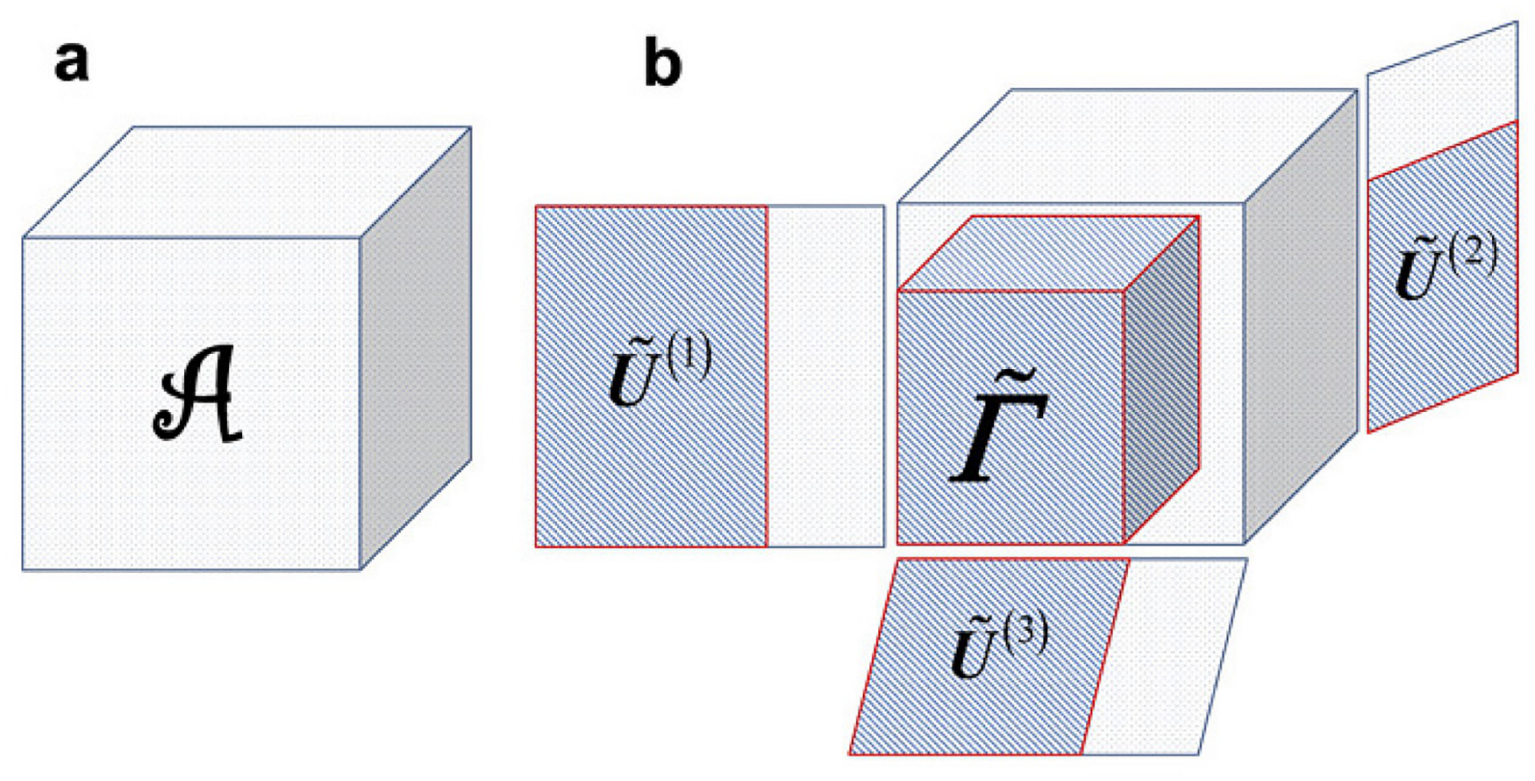
The truncated HOSVD of a 3-mode tensor. Part **a** shows the original tensor A, and Part **b** displays the concept of a truncated HOSVD of A.

For a tensor $A\in R^{I_{1}\times I_{2}\times \ldots \times I_{N}}$, the *n*-rank of $A(rank_{n}(A))$ is defined as the column rank of **A**_(*n*)_. Let $r_{n}=rank_{n}(A)$, (*n* = 1, …, *N*), then A has the rank of (*r*_1_, *r*_2_, …, *r*_*N*_). Inspired by the rank-*l* (*l* < *r*) SVD approximation of matrix **A** in Eq (7), the singular-value truncation also can be used to reveal effective signals and reduce noises in a tensor [23]. Accordingly, a portion of singular values *Σ*^(*n*)^ in Eq (22) will be adopted. Thus, we can define a truncated HOSVD depending on the matrix format as follows, *for a tensor A, its decomposition of rank-$\left({\tilde{r}}_{1},{\tilde{r}}_{2},\ldots ,{\tilde{r}}_{N}\right)$, with ${\tilde{r}}_{n}<r_{n}=rank_{n}\left(A\right)$ for at least one mode (n), is called a truncated HOSVD* [40]. This concept for a 3-mode tensor is shown in Fig 1.

**Fig 2:**
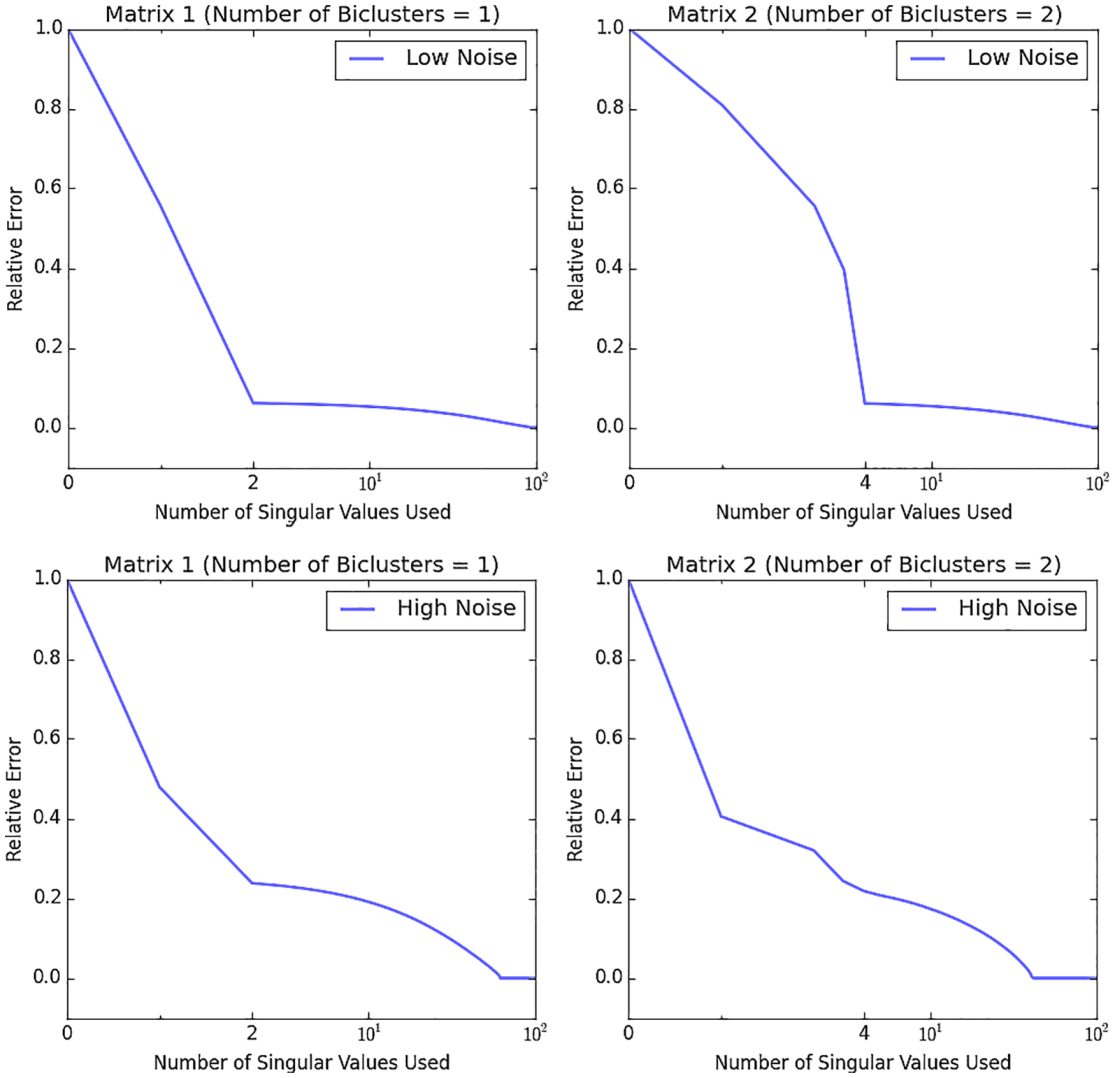
Derivation of the optimal rank. According to the relative error curve with different number of singular values used, the optimal rank of the Matrix 1 and Matrix 2 are 2 and 4, respectively.

The optimal rank $\left({\tilde{r}}_{1},{\tilde{r}}_{2},\ldots ,{\tilde{r}}_{N}\right)$ of a tensor can be determined from a compromise between accuracy and number of singular values used. Because we use the unfolded matrices to determine the row indices of co-clusters, we find the optimal rank ${\tilde{r}}_{n}$ of each unfolded matrix **A**_(*n*)_ and the optimal rank of the tensor is then (${\tilde{r}}_{1}$, ${\tilde{r}}_{2}$, …, ${\tilde{r}}_{N}$). The accuracy of a matrix $\tilde{A}$ is defined using the Frebenius norm as $acc=\frac{∥A-\tilde{A}{∥}_{F}^{2}}{{∥A∥}_{F}^{2}}$, where $\tilde{A}$ is reconstructed from SVD after truncation of mall singular values. The relative error is defined as 1−*acc*. Fig 2 presents the relative error curves for two examples **A** and $\tilde{A}$ with different numbers of singular values used. The two 2D synthetic matrices of size 100 × 100, which are similar to Matrix 1 and Matrix 2 discussed below **Proposition 3**, are embedded with one and two additive biclusters of size 10 × 10, respectively. Each additive bicluster is produced from a seed column of random numbers distributed in a normal distribution **N**(0, *ζ*^2^) (*ζ* = 6 for low nosie level and *ζ* = 1.25 for high noise level) and each of the other 9 columns is produced by adding to the seed column a random number with the standard normal distribution. The background of each matrix also contains random numbers with the standard normal distribution. As shown in Fig 2, the corner point corresponds to the optimal rank (2 and 4) of the two 2D synthetic matrices. As noise increases, the relative error curve becomes smooth and the corner point disappears. Through many simulation experiments, we find that a threshold at 20% relative error can be used to find the optimal rank value.

#### Linear Grouping Algorithm (LGA)

As discussed in Section “Biclustering Based on Singular Value Decomposition (SVD) and Clustering in Singular Vector Matrices”, bicluster searching in a 2D matrix **A** can be transformed into a hyperplane detection problem in the singular vector matrices produced by SVD, which can be generalized to a high-order tensor A. In detail, a co-cluster in A can be represented by the biclusters along each mode, and a series of linear relations among the HOSVD-generated singular vectors can be accordingly inferred as below,

$$\begin{array}{c} \left\{\begin{array}{c} \alpha_{11}u_{1}^{(1)c}+\alpha_{12}u_{2}^{(1)c}+\ldots +\alpha_{1{\tilde{r}}_{1}}u_{{\tilde{r}}_{1}}^{(1)c}+\beta_{1}=0\\ \alpha_{21}u_{1}^{(2)c}+\alpha_{22}u_{2}^{(2)c}+\ldots +\alpha_{2{\tilde{r}}_{2}}u_{{\tilde{r}}_{2}}^{(2)c}+\beta_{2}=0\\ \vdots \\ \alpha_{N1}u_{1}^{(N)c}+\alpha_{N2}u_{2}^{(N)c}+\ldots +\alpha_{N{\tilde{r}}_{N}}u_{{\tilde{r}}_{N}}^{(N)c}+\beta_{N}=0 \end{array}\right. \end{array}$$ (23)

where the co-cluster is decomposed as $A_{c}=T_{c}\times_{1}{\tilde{U}}^{(1)c}\times_{2}{\tilde{U}}^{(2)c}\ldots \times_{N}{\tilde{U}}^{(N)c}$.

Eq (23) represents a group of hyperplanar (linear) relations in a multi-dimensional space, which can be named *hyperplanar co-clusters*. Importantly, similar to the 2D case, these hyperplanar relations shed light on the multi-dimensional co-cluster identification in tensors [5, 13, 36, 37, 39].

Specifically in our work, the *linear grouping algorithm* (LGA) was adopted to reveal the linear relations embedded in the singular vector matrices, which were generated by a truncated HOSVD. This model follows the linear patterns among the involved vectors [5]. Originally, LGA clustered data points by fitting a mixture of linear regression models [42], and later an evolved model based on an orthogonal regression approach was proposed, which provided favorable performances in applications with outliers [5]. Recently, this model has been improved to a robust linear clustering method [43]. A simple procedure of LGA can be described as **Algorithm 1**.

**Algorithm 1** Procedure of LGA for a group of vectors $U_{I_{n}\times {\tilde{r}}_{n}}$

 **procedure** LGA $\left(U_{I_{n}\times {\tilde{r}}_{n}}={\left[u_{1},u_{2},\ldots ,u_{I_{n}}\right]}^{T},K\right)$

   **Step 1:** Scale the variables through dividing them by the standard deviation,

   ${\tilde{U}}_{I_{n}\times {\tilde{r}}_{n}}={\left[{\tilde{u}}_{1},{\tilde{u}}_{2},\ldots ,{\tilde{u}}_{I_{n}}\right]}^{T}$,

    where $\left\{ \begin{array}{c} {\tilde{u}}_{i}=\frac{u_{i}}{s_{i}}\\ s_{i}=\sqrt{\frac{1}{d-1}\left(u_{i}-{\bar{u}}_{i}\right){\left(u_{i}-{\bar{u}}_{i}\right)}^{T}}\\ i=1,2,\ldots ,I_{n} \end{array}\right.$

   **Step 2:** Select *K* random sub-samples of size ${\tilde{r}}_{n}$,

    $G^{0}=\left\{g_{1}^{0},g_{2}^{0},\ldots ,g_{K}^{0}\right\}$

    where $\left\{ \begin{array}{c} g_{k}^{0}=\left[{\tilde{u}}_{k1}^{0},{\tilde{u}}_{k2}^{0},\ldots ,{\tilde{u}}_{k{\tilde{r}}_{n}}^{0}\right]\\ {\tilde{u}}_{ki}^{0}\in {\tilde{U}}_{I_{n}\times {\tilde{r}}_{n}} \end{array}\right.$

   **Step 3:** Loop

    **for** *j* = 0, 1, …, *J* **do**

1. Initialize *K* orthogonal regression hyperplanes $H^{j}=\left\{h_{1}^{j},h_{2}^{j},\ldots ,h_{K}^{j}\right\}$ by fitting the samples in $G^{j}=\left\{g_{1}^{j},g_{2}^{j},\ldots ,g_{K}^{j}\right\}$, resulting in $h_{k}^{j}=\{\left({\hat{\alpha }}_{k},{\hat{\beta }}_{k}\right)|{\hat{\alpha }}_{k}^{T}{\tilde{u}}_{ki}^{j}-{\hat{\beta }}_{k}=0,{\tilde{u}}_{ki}^{j}\in g_{k}^{j},∥{\hat{\alpha }}_{k}∥=1,i=1,2,\ldots ,{\tilde{r}}_{n}\}\;\left(k=1,2,\ldots ,K\right)$.
2. Compute the distance between each hyperplane $h_{k}^{j}$ and each sample ${\tilde{u}}_{i}$, $d_{ik}^{j}=distance\left({\tilde{u}}_{i},h_{k}^{j}\right)=\left|{\hat{\alpha }}_{k}^{T}{\tilde{u}}_{i}-{\hat{\beta }}_{k}\right|$, where $\left\{ \begin{array}{c} k=1,2,\ldots ,K\\ i=1,2,\ldots ,I_{n} \end{array}\right.$.
3. Form *K* groups for ${\tilde{u}}_{i}$, $G^{j+1}=\left\{g_{1}^{j+1},g_{2}^{j+1},\ldots ,g_{K}^{j+1}\right\}$, where ${\tilde{u}}_{i}\in g_{k}^{j+1}$ if $k=argmin_{k}\left(d_{ik}^{j}\right)$.
4. Compute the evaluation function for each iteration, $D^{j}=\sum_{k=1}^{K}\sum_{{\tilde{u}}_{i}\in g_{k}^{j+1}}d_{ik}^{j}$.

   **end for** *D*^*j*^ reaches the minimum *D*^*opt*^.

   Return **G**^*opt*^

 **end procedure**

The purpose of applying the LGA algorithm is to find the row indices of each co-cluster along each mode of a tensor from the corresponding unfolded matrix, as discussed above. This is done by detecting hyperplanes formed by some but not all points (*u*_*m*1_, *u*_*m*2_, …, *u*_*md*_) (*m* = 1, 2, …, *I*_*n*_) in the left singular vector space along each mode. Initially, we choose random points to compute the coefficients of hyperplanes. Then we assign each point to the closest hyperplane. This process is repeated to improve the result. The procedure is similar to the K-means algorithms. The difference is that we deal with hyperplanes while the K-means algorithm involves cluster centers only. We consider a point is on a hyperplane if its distance to the hyperplane is smaller than a pre-specified threshold, which is determined experimentally. From a set of points on the same hyperplane, we can then find the row indices of a corresponding co-cluster. All co-clusters detected from the hyperplanes are subject to an evaluation and elimination procedure discussed below.

#### Multi-dimensional Co-clustering in Tensors Based on HOSVD and LGA

**Fig 3:**
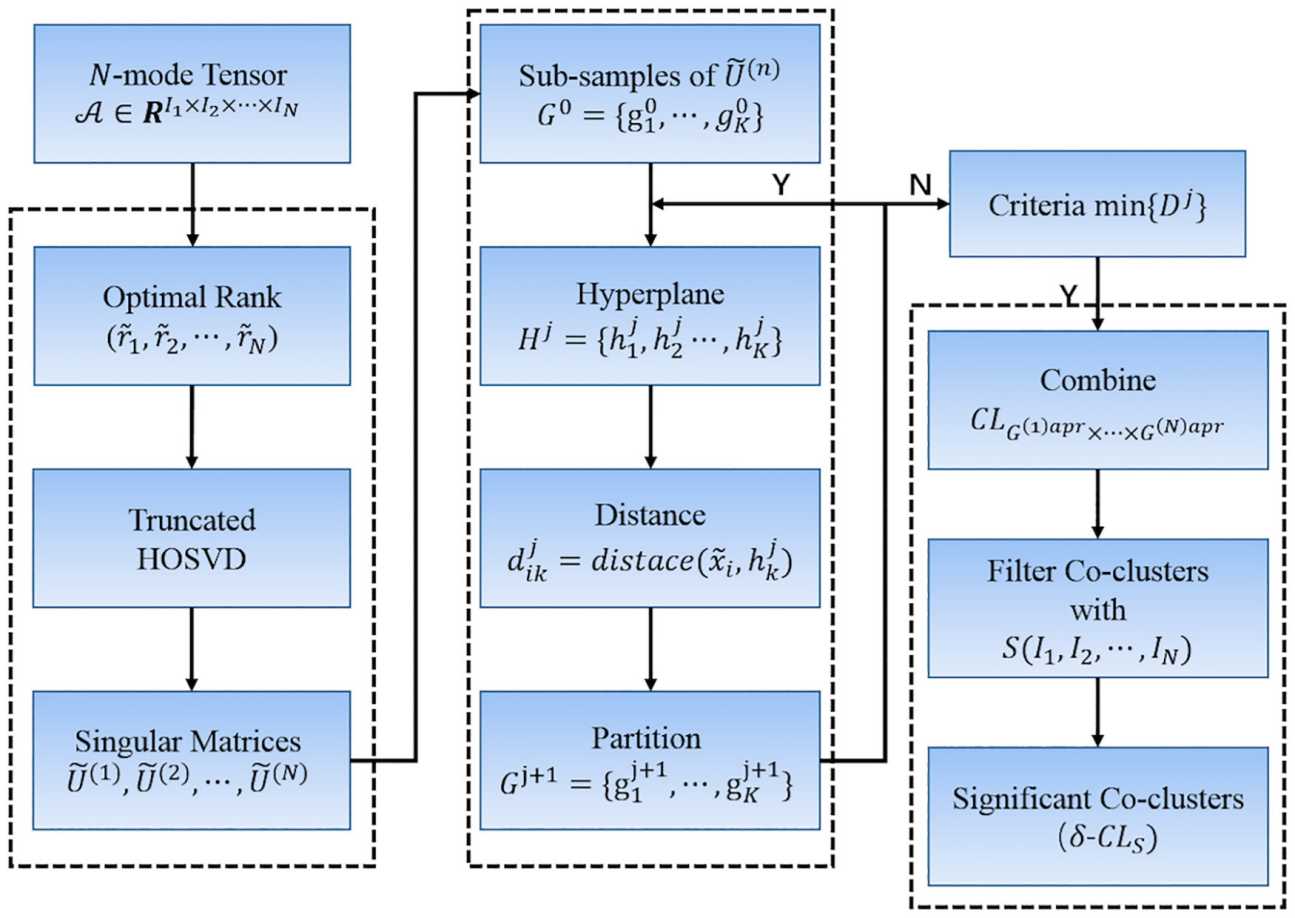
Block diagram of our co-clustering method based on hyperplane detection in singular vector spaces (*HDSVS*). In the left-hand side, the flow for a truncated HOSVD is shown. The LGA module is presented in the middle. The ranking procedure based on a scoring function, for revealing significant co-clusters (*δ*-*CL*s) in a tensor, is listed in the right-hand side.

HOSVD decomposes an *N*-mode tensor A into a core tensor and singular vector matrices **U**^(*n*)^ along all modes. Hyperplanes embedded in each truncated singular vector matrix were revealed by LGA, based on the natural linear patterns among the vectors. Through combining the products along each mode, the high-order co-clusters in this tensor were successfully identified. To further filter such co-clusters, those having a score (Eq (20)) smaller than or equal to *δ*, namely *S*(**I**_1_, **I**_2_, …, **I**_*N*_) ≤ *δ* (*δ* > 0), were extracted and regarded as more significant. A co-cluster is eliminated if it is a part of or identical to another co-cluster. The block diagram of our co-clustering method based on hyperplane detection in singular vector spaces (***HDSVS***) is shown in Fig 3.

## Experiment Results

To verify ***HDSVS***, several data sets, including multiple synthetic and biological tensors, were used in the experiments. Two synthetic tensors were constructed with increased noise and overlapped degree, to evaluate the effects of noise and overlapping complexity to the co-cluster identification. Two biological tensors were from *gene expression data from 12 multiple sclerosis patients under an IFN-β therapy* [44, 45] and *spatial/temporal lineage tracing data of embryonic cells in a crowd of Caenorhabditis elegans* [34, 46], and were used to test the performance of our method in practical problems. ***HDSVS*** can be successfully applied to co-clustering in high-order tensors. However, because most existing methods are designed for 2D data, we conducted comparisons of ***HDSVS*** with other methods using only matrices or second-order tensors in Section “Experiment Comparisons with Other Methods Using 2D Synthetic Data and 2D Yeast Gene Expression Data”. Specifically, 2D synthetic tensors generated based on well-published principles [47] and a 2D yeast gene expression tensor [20] were adopted to compare the performance and robustness of our method with those of existing methods.

### Evaluation of Noise and Overlapping Effects in Co-cluster Identification Using Synthetic Tensors

A matching scoring, generated by the Jaccard coefficient [14], was first defined to evaluate the agreement between a detected co-cluster and the true one. Let $CL_{1}=\left(G_{1}^{1},G_{2}^{1},\ldots ,G_{N}^{1}\right)$ and $CL_{2}=\left(G_{1}^{2},G_{2}^{2},\ldots ,G_{N}^{2}\right)$ be two co-clusters in a tensor $A\in R^{I_{1}\times I_{2}\times \ldots \times I_{N}}$, where $G_{i}^{j}\subseteq I_{i}\;\left(i=1,2,\ldots ,N;j=1,2\right)$ is a subset of the *i*th dimension of A. The matching score can be expressed as follows,

$$\begin{array}{c} MS\left(CL_{1},CL_{2}\right)={\text{max}}_{CL_{1}}{\text{max}}_{CL_{2}}\frac{\sum_{i=1}^{N}\left|G_{i}^{1}\cap G_{i}^{2}\right|}{\sum_{i=1}^{N}\left|G_{i}^{1}\cup G_{i}^{2}\right|} \end{array}$$ (24)

Further, we denote a true co-cluster as *CL*_*true*_ and a detected one as *δ*-*CL*, then a larger value of *MS*(*CL*_*true*_, *δ*-*CL*) represents a better detection. Based on such matching scores, effects of noise and overlapping complexity on the co-cluster identification will be discussed, using the two synthetic tensors.

**Fig 4:**
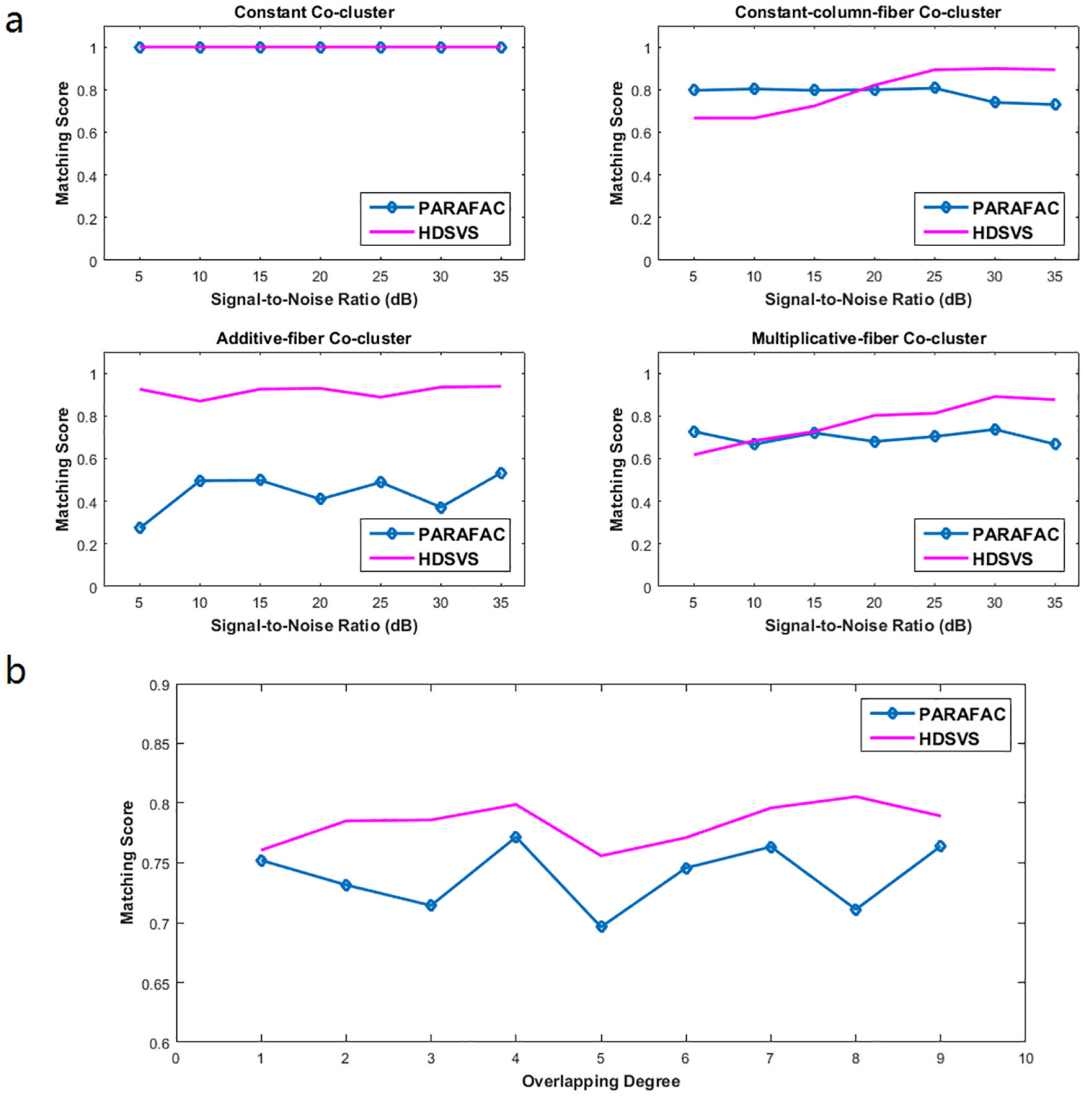
Effects of noise and overlapping complexity on co-cluster identification in two synthetic tensors. (a) Matching scores between true co-cluaters and the detected ones, with different SNRs. (b) Matching scores between two overlapping co-clusters and the true ones, with various overlapping degrees.

In the first case, four types of *CL*_*true*_ (10 × 10 × 10), constant, constant-column-fiber, additive-fiber and mulitplicative-fiber co-clusters, were embedded into a 3D tensor $A_{1}$ (100 × 100 × 100), whose background was generated based on the standard normal distribution. The four types of co-clusters were generated as follows:

1. constant co-cluster, i.e. {*a*_*ijk*_ = 2 ∣ *i* ∈ **I**, *j* ∈ **J**, *k* ∈ **K**};
2. constant-column-fiber co-cluster, i.e. {**a**_**I***jk*_ = *μ*_*jk*_**1**_**I**_ ∣ *j* ∈ **J**, *k* ∈ **K**}, where *μ*_*jk*_ was randomly selected from *U*(−2, 2);
3. additive-fiber co-cluster, i.e. {**a**_**I***jk*_ = *μ*_*jk*_ **1**_**I**_ + **a**_**I**(1)_ ∣ *j* ∈ **J**, *k* ∈ **K**}, where **a**_**I**(1)_ is the first fiber of the co-cluster, *μ*_*jk*_ and each value of **a**_**I**(1)_ were randomly selected from *U*(−2, 2);
4. multiplicative-fiber co-cluster, i.e. {**a**_**I***jk*_ = *μ*_*jk*_ **a**_**I**(1)_ ∣ *j* ∈ **J**, *k* ∈ **K**}, where **a**_**I**(1)_ is the first fiber of the co-cluster, where *μ*_*jk*_ and each value of **a**_**I**(1)_ were randomly selected from *U*(−2, 2);

Then Gaussian white noise with different signal-to-noise ratios (SNRs) was generated to degrade *CL*_*true*_. The proposed ***HDSVS*** algorithm and PARAFAC with sparse latent factors [29] was then applied to the noisy tensors, after which *MS*(*CL*_*true*_, *δ*-*CL*) was calculated. The experiment was performed 100 times for each method and the matching scores from all experiments were averaged to obtain the final score for comparison. The matching scores corresponding to various SNRs are summarized in Fig 4a. For the constant co-cluster, the matching score of ***HDSVS*** and PARAFAC are 1 for all SNRs. PARAFAC performs better than ***HDSVS*** when the SNR is low for the constant-column-fiber (*SNR* ≤ 15) and the multiplicative-fiber (*SNR* ≤ 5) co-cluster. However, as the SNR increases, ***HDSVS*** has higher matching scores than PARAFAC for the constant-column-fiber (*SNR* ≥ 20) and the multiplicative-fiber (*SNR* ≥ 10) co-cluster. HDSVS is much better than PARAFAC for additive-fiber co-clusters. As discussed above, constant, constant-column and multiplicative biclusters have rank 1, while additive biclusters have rank 2. Our proposed HDSVS algorithm is especially effective for additive co-clusters because the hyperplane model fits their linear structures well. The PARAFAC based co-clustering method relies on the K-means clustering formulation and can only work well with co-clusters that can be represented by their centers, corresponding to structures of rank 1.

Likewise, a 3D tensor $A_{2}$ (100 × 100 × 100) with two overlapping co-clusters were generated. The experiment was also performed 100 times. Each time, the types of two overlapping co-clusters were chosen randomly. For simplicity, merely the overlapped cubic patterns were considered in the evaluation, and thus the overlapping degree *v* can be defined as the size of overlapped cubes in each dimension (0 ≤ *v* ≤ 9). As shown in Fig 4b, both methods have reasonably good performance for detecting overlapping co-clusters. However, for all overlapping degrees, HDSVS performs consistently better than PARAFAC.

### Co-cluster Identification in Gene Expression Data from Sclerosis Patients under an IFN- *β* Therapy

A 3D tensor generated from the gene expression data of multiple sclerosis patients, who accepted a treatment of IFN-*β* injection, is discussed here. Twelve patients with western European ancestry, including eight females and four males (average age of 36.4), were recruited in this study. Fifteen milliliters of EDTA blood sample (at peripheral venous) were drawn from each patient, respectively at the baseline day and 2 days, 1 month, 1 year and 2 years after the initiation of an IFN-*β* therapy (http://link.springer.com/article/10.1007/s12035-013-8463-1/fulltext.html) [44, 45].

**Fig 5:**
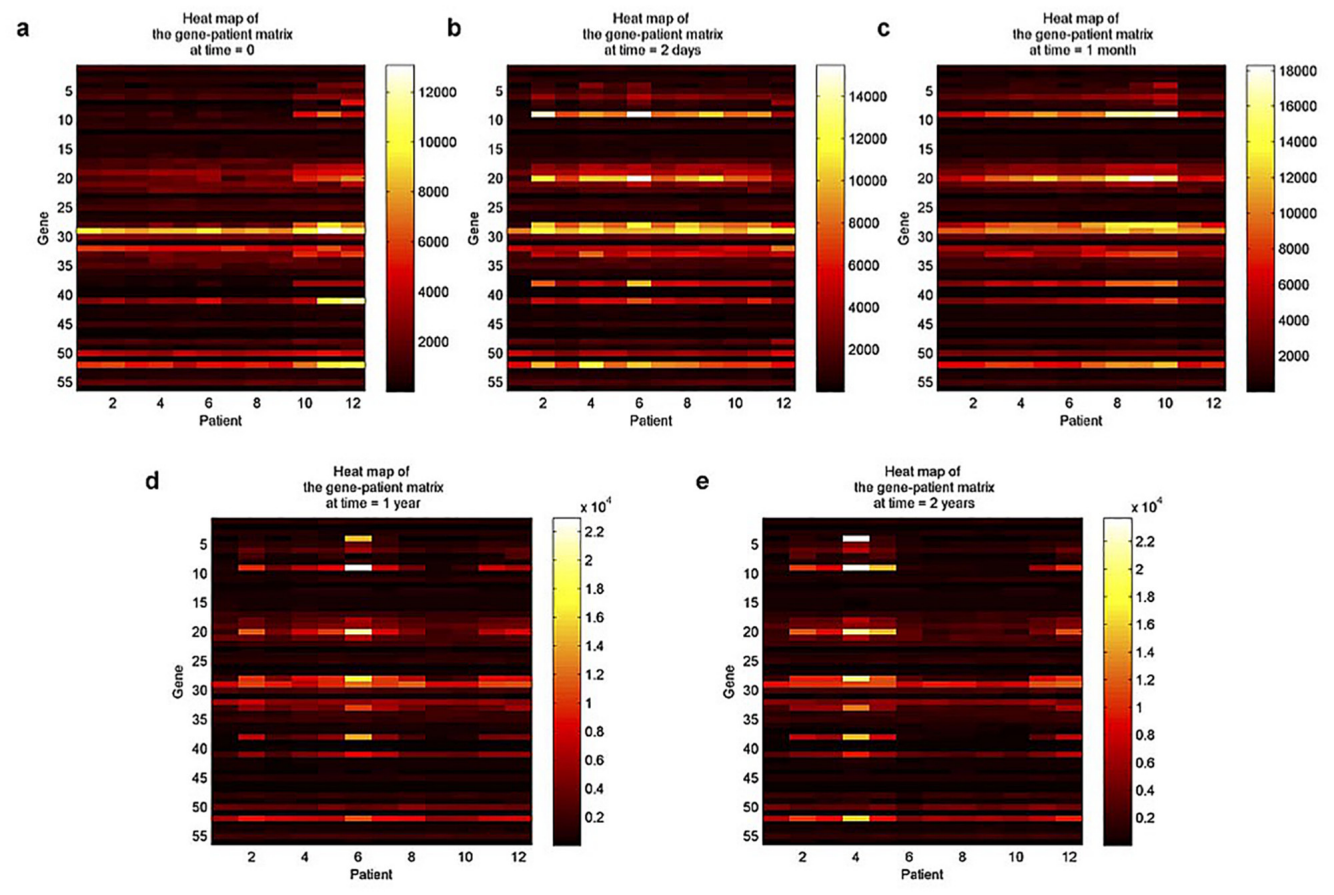
Heat maps for gene×patient matrices at fixed time points. (a) to (e), Scenarios for gene×patient heat maps corresponding to the baseline day, and 2 days, 1 month, 1 year and 2 years after the initiation of an IFN-*β* therapy.

In detail, the gene expression data can be represented by a 3D tensor (gene×patient×time) of size 18862 × 12 × 5. Considering the IFN-*β* therapy, we only kept the therapy-related genetic pathways (56 genes), leading to a simplified tensor of size 56 × 12 × 5. Regarding each gene×patient matrix (time is fixed) as a layer, Fig 5 shows the heat maps for these five layers.

**Fig 6:**
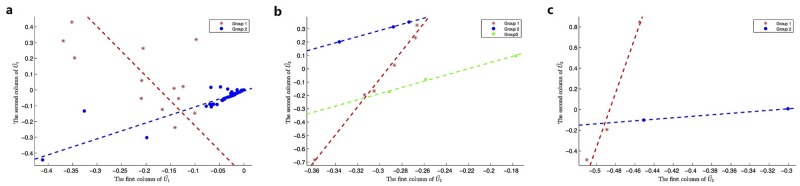
The linear patterns embedded in the 2D singular-vector space. (a) to (c), The linear groups along the directions of first two singular vectors of **U**^(*n*)^ (*n* = 1, 2, 3), respectively.

**Fig 7:**
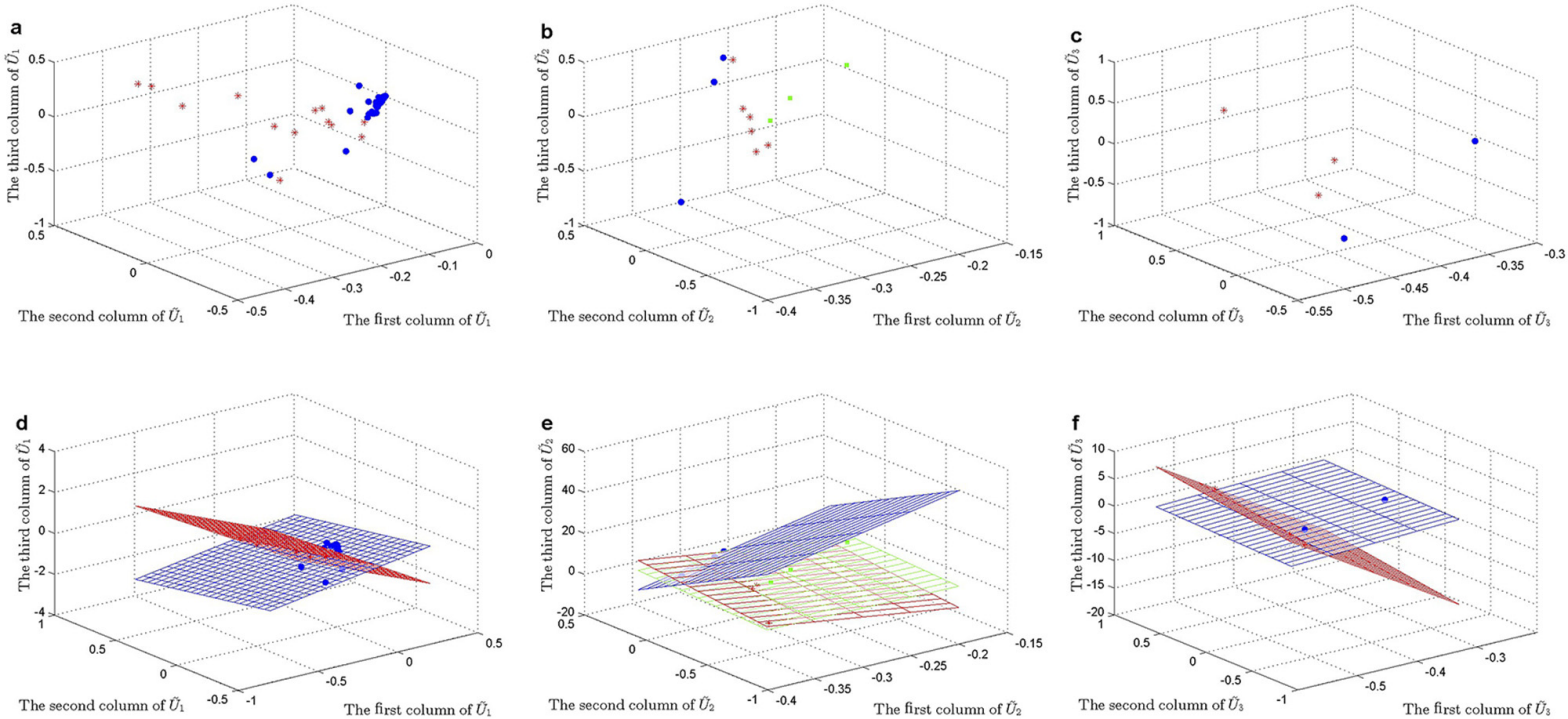
Linear patterns embedded in the 3D singular-vector space of the gene×patient×time tensor. (a) to (c), The scatter plots along the directions of first three singular vectors of **U**^(*n*)^ (*n* = 1, 2, 3), respectively. (d) to (f), Linear or planar patterns of the 3D-points in (a) to (c).

***HDSVS*** was applied on this simplified tensor A. Its core tensor T and singular vector matrices **U**^(*n*)^ (*n* = 1, 2, 3) can be extracted by HOSVD, while considering noises, a truncated HOSVD $\tilde{A}$ was implemented ($\tilde{T}$ and ${\tilde{U}}^{\left(n\right)}$). Specifically, the optimal rank for the truncated HOSVD is (2, 4, 4), which was derived based on the method discussed in Section “HOSVD” and Fig 2. Once the truncated HOSVD was obtained, LGA was used for the bicluster-detection along each mode (${\tilde{U}}^{\left(n\right)}$). For example, the 56 points in ${\tilde{U}}^{\left(1\right)}$ can be linearly grouped into two patterns, and similar patterns can be derived for ${\tilde{U}}^{\left(2\right)}$ and ${\tilde{U}}^{\left(3\right)}$. Such linear patterns, at the first two dimensions of each ${\tilde{U}}^{\left(n\right)}$ (or **U**^(*n*)^), are shown in Fig 6a to 6c, respectively. As a supplementary study, the first three dimensions of each ${\tilde{U}}^{\left(n\right)}$ (or **U**^(*n*)^) are separately plotted in Fig 7a to 7c. Further, the linear or planar patterns of these points in the 3D space are displayed in Fig 7d to 7f. The planar structures consistently validates the grouping or clustering capability of ***HDSVS***.

As shown in Fig 6, the 56 genes are divided into two linear groups denoted as **E**^*i*^ (*i* = 1, 2), the 12 patients correspond to three groups **P**^*j*^ (*j* = 1, 2, 3), and the 5 time points represent two groups **T**^*k*^ (*k* = 1, 2). Accordingly, we combine these indexes to build a co-cluster as follows,

$$\begin{array}{c} \begin{array}{c} CL_{ijk}=\left\{\left(G_{1}^{i},G_{2}^{j},G_{3}^{k}\right)\right\}\\ =\{\left(E^{i},P^{j},T^{k}\right)|i=1,2;j=1,2,3;k=1,2\} \end{array} \end{array}$$ (25)

To refine the findings, significant *δ*-*CL*s (Eq (19)) were extracted. *CL*_121_, including 13 genes, 6 patients, and 2 time points, finally stood out with *δ* = 0.156.

**Fig 8:**
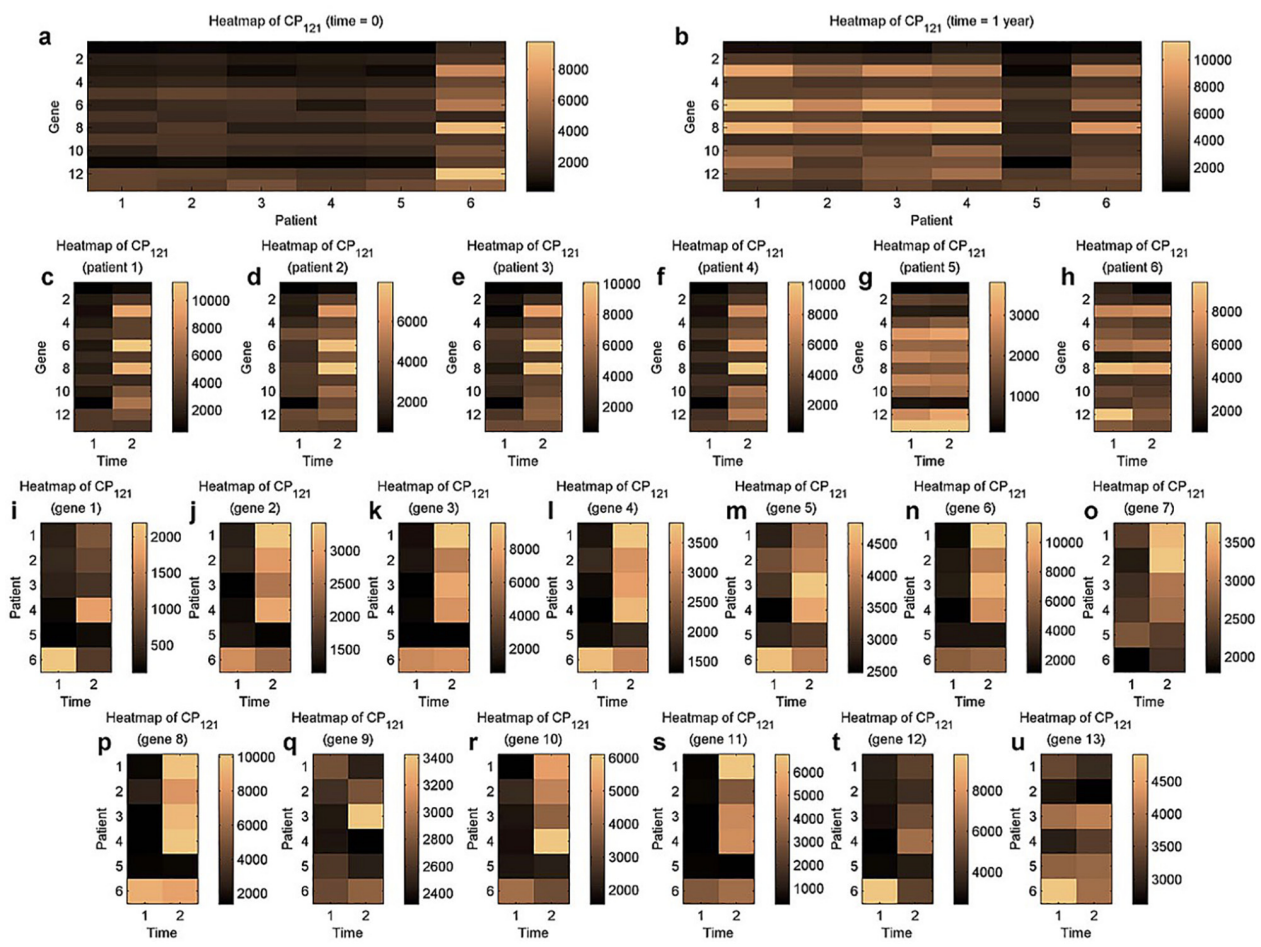
Heat maps (along each two modes) of the significant co-cluster *CL*_121_ in the gene×patient×time tensor. (a) and (b), Heat maps of the gene×patient matrix at the baseline time and at time = 1 year. (c) to (h), Heat maps of the gene×time matrices for the 6 patients. (i) to (u), Heat maps of the patient×time matrices for the 13 genes.

To profile this co-cluster, we observed it along each two modes and now present the heat maps in Fig 8. Fig 8a and 8b display the gene×patient matrices at the baseline time and 1 year after the IFN-*β* therapy. In Fig 8c through Fig 8h, the gene×time matrices for the 6 involved patients are shown. Here we can detect the similar patterns among the first four patients (Fig 8c to 8f), roughly defining a constant-lateral-slice or additive-lateral-slice co-cluter. Additionally, the patient×time matrices for the 13 genes in this co-cluster are presented in Fig 8i to 8u. Interestingly, an L-shape is revealed in the majority of these figures, and this similarly leads to the formation of an additive-horizontal-slice or multiplicative-horizontal-slice co-cluster. All these results have attested the reliability of our algorithm.

The biological significance of *CL*_121_ was further analyzed according to genetic pathways and ***GO*** annotations [33]. Specifically, important contribution of the 13 genes (CXCL10, EIF2AK2, IFIT1, IRF7, IRF9, ISG15, ISG20, MX1, NFKB1, OAS1, RSAD2, STAT1, TLR8) to the biological processes in ***GO*** has long been elucidated (Table 1). As reported in [48, 49], the *immune system process* (GO: 0002376) was consistently annotated, verifying the enrichment of its three sub-terms, *immune effector process*, *defense response to virus* and *response to virus* in our detected co-cluster.

**Table 1:** Biological processes regulated by the 13 annotated genes in the significant co-cluster *CL*_121_ (identified in the gene×patient×time tensor).

GO term	Description	*P*-value	Enrichment (N, B, n, b)
GO:0002252	Immune effector process	8.81E-5	2.07 (56,25,13,12)
GO:0051607	Defense response to virus	1.17E-4	2.26 (56,21,13,11)
GO:0045069	Regulation of viral genome replication	2.12E-4	3.35 (56,9,13,7)
GO:0045071	Negative regulation of viral genome replication	2.12E-4	3.35 (56,9,13,7)
GO:0009615	Response to virus	2.77E-4	1.91 (56,27,13,12)
GO:0048525	Negative regulation of viral process	6.28E-4	3.02 (56,10,13,7)
GO:0050792	Regulation of viral process	6.28E-4	3.02 (56,10,13,7)

Moreover, the genetic pathways of the 13 genes were analyzed (Table 2). These pathways were mostly inferred from previous studies [44, 45]. Interestingly, the enrichment of *bone remodeling pathway* is shown in our analysis, with a small *p*-value of 2.3E-4 in Table 2. This finding leads to sufficient biological evidences of correlating *bone remodeling* with the IFN-*β* treatment for sclerosis patients.

**Table 2:** Genetic pathways of the 13 annotated genes in the significant co-cluster *CL*_121_ (identified in the gene×patient×time tensor).

Pathway annotated	Term	*P*-value	Benjamini adjusted *p*-value
BIOCARTA	Bone remodelling	2.3E-4	1.0E-2
BIOCARTA	IFN-*α* signaling pathway	1.9E-2	3.5E-1
BIOCARTA	Double stranded RNA induced gene expression	1.9E-2	3.5E-1
BIOCARTA	Inactivation of Gsk3 by AKT causes accumulation of b-catenin in alveolar macrophages	5.9E-2	6.0E-1
BIOCARTA	Toll-like Receptor Pathway	7.5E-2	5.9E-1
KEGG PATHWAY	Toll-like receptor signaling pathway	2.1E-6	4.3E-5
KEGG PATHWAY	RIG-I-like receptor signaling pathway	5.1E-5	5.1E-4
KEGG PATHWAY	Cytosolic DNA-sensing pathway	1.7E-3	1.1E-2
KEGG PATHWAY	Chemokine signaling pathway	1.8E-2	8.8E-2
KEGG PATHWAY	Pancreatic cancer	8.2E-2	2.9E-1

Common characteristics of the patients [48], such as a shorter disease duration, a lower EDSS score and an easier relapse, in *CL*_121_ were further revealed, leading to an effective profile of their disease progression. Overall, our algorithm can be beneficial to personalized therapy design and new drug discovery in the treatments of sclerosis patients.

### Co-clustering of Embryonic Cell Cycles in the Lineages of C. Elegans

**Fig 9:**
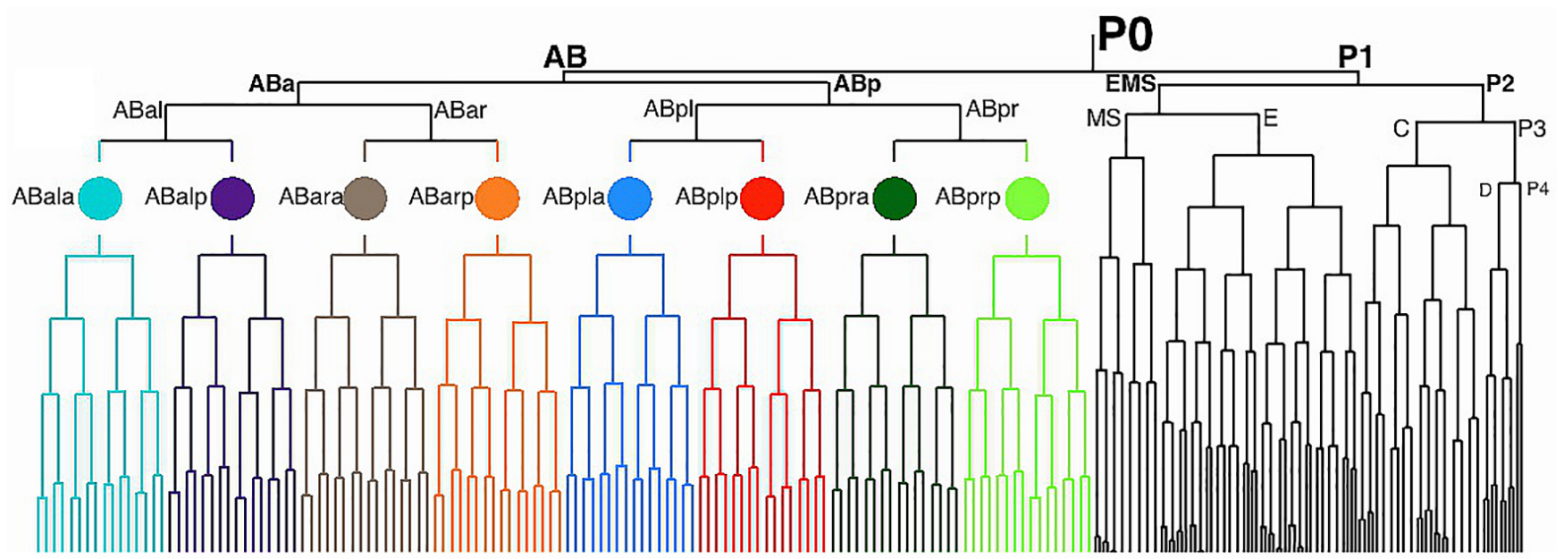
Lineage tracing of C. elegans at the 200-cell stage.

Recently, the techniques of live-cell imaging microscopy and fluorescent tagging have developed rapidly. These techniques have been broadly used in observing gene expression, nuclei movement and nuclei division, during the embryogenesis of a single cell [34, 38, 46]. An example of the lineage tracing of C. elegans can be found in Fig 9.

The length of a cell cycle in an organism is important in the tracing of an embryonic cell lineage. For different organisms and cells, the length varies significantly. Depending on well-reported protocols in [46], the cell cycle lengths of ∼300 C. elegans embryos were evaluated by perturbing their 1219 genes in [49] (http://phenics.icts.hkbu.edu.hk/). For simplicity, the 8-cell stage in the AB branch was regarded as our founder-cell stage [34], and 14 descendants of each founder cell were studied. As a summary, a gene×descendant×founder tensor of size 1219 × 14 × 8 was constructed. Importantly, identification of co-clusters in this tensor can lead to the derivation of cell fates in the C. elegans lineage.

**Fig 10:**
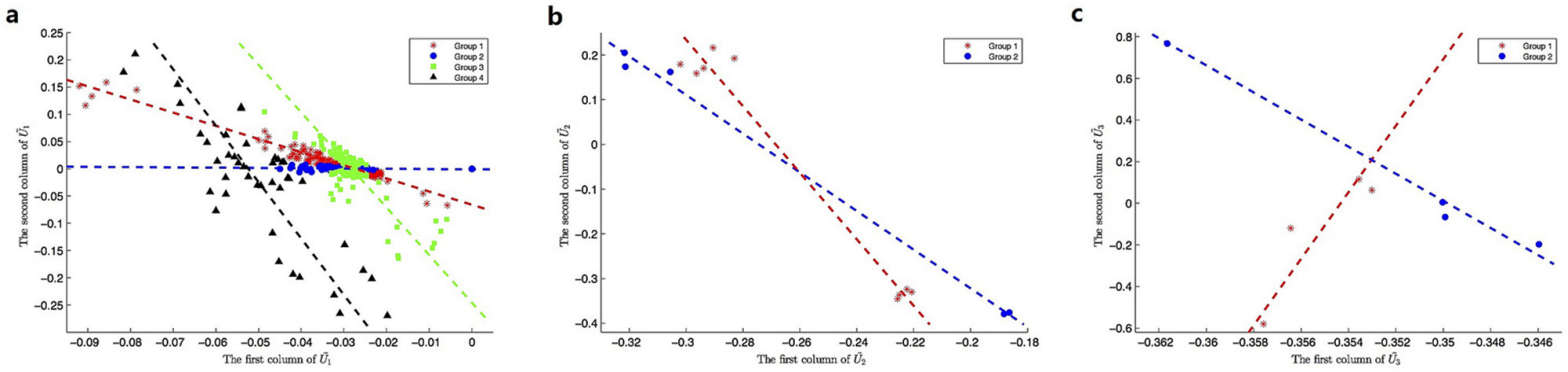
The linear patterns embedded in the 2D singular-vector space. (a) to (c), The linear groups along the directions of first two singular vectors of **U**^(*n*)^ (*n* = 1, 2, 3), respectively.

**Fig 11:**
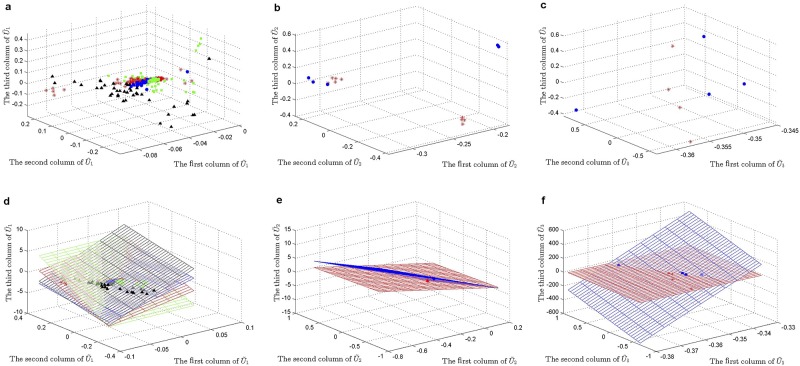
Linear patterns embedded in the 3D singular-vector space for the lineage tracing data of C. elegans. (a) to (c), The scatter plots along the directions of first three singular vectors of **U**^(*n*)^ (*n* = 1, 2, 3), respectively. (d) to (f), Linear or planar patterns of the 3D-points in (a) to (c).

Similar to the preceding section, the optimal rank of (2, 5, 8) was derived for the truncated HOSVD. Linear patterns (LGA) in ${\tilde{U}}^{\left(n\right)}$ were separately displayed in Fig 10a to 10c, where the first two column vectors were used as representatives. Meanwhile, the first three dimensions of each ${\tilde{U}}^{\left(n\right)}$ (Fig 11a to 11c) and their planar patterns in the 3D space are separately displayed in Fig 11d to 11f.

Specifically, 4, 2 and 2 clusters in the three modes were detected and are shown in Table 3. Here, the two groups of founder cells (**F**) are consistently traced to their mother cells (ABal/ABpl and ABar/ABpr). Furthermore, all the cells in **D**^2^ are terminal cells, and only three terminal cells (*aaa, *paa and *pap) are distributed to **D**^1^. Compared to ancestors, terminal cells at the same stage were reasonably grouped together, as earlier cell cycles had evolved to different lengths after multiple cell divisions.

**Table 3:** The linear groups in different modes of the gene×descendant×founder tensor (“*” denotes a founder cell).

Features modes		The corresponding linear groups
Perturbed genes (**E**)	**E**^1^	The number of genes in the group is 42
	**E**^2^	The number of genes in the group is 729
	**E**^3^	The number of genes in the group is 379
	**E**^4^	The number of genes in the group is 69
descendant cells (**D**)	**D**^1^	“*a” “*aa” “*aaa” “*ap” “*p” “*pa” “*paa” “*pap” “*pp”
	**D**^2^	“*aap” “*apa” “*app” “*ppa” “*ppp”
founder cells (**F**)	**F**^1^	“*ABala*” “*ABalp*” “*ABpla*” “*ABplp*”
	**F**^2^	“*ABara*” “*ABarp*” “*ABpra*” “*ABprp*”

**Fig 12:**
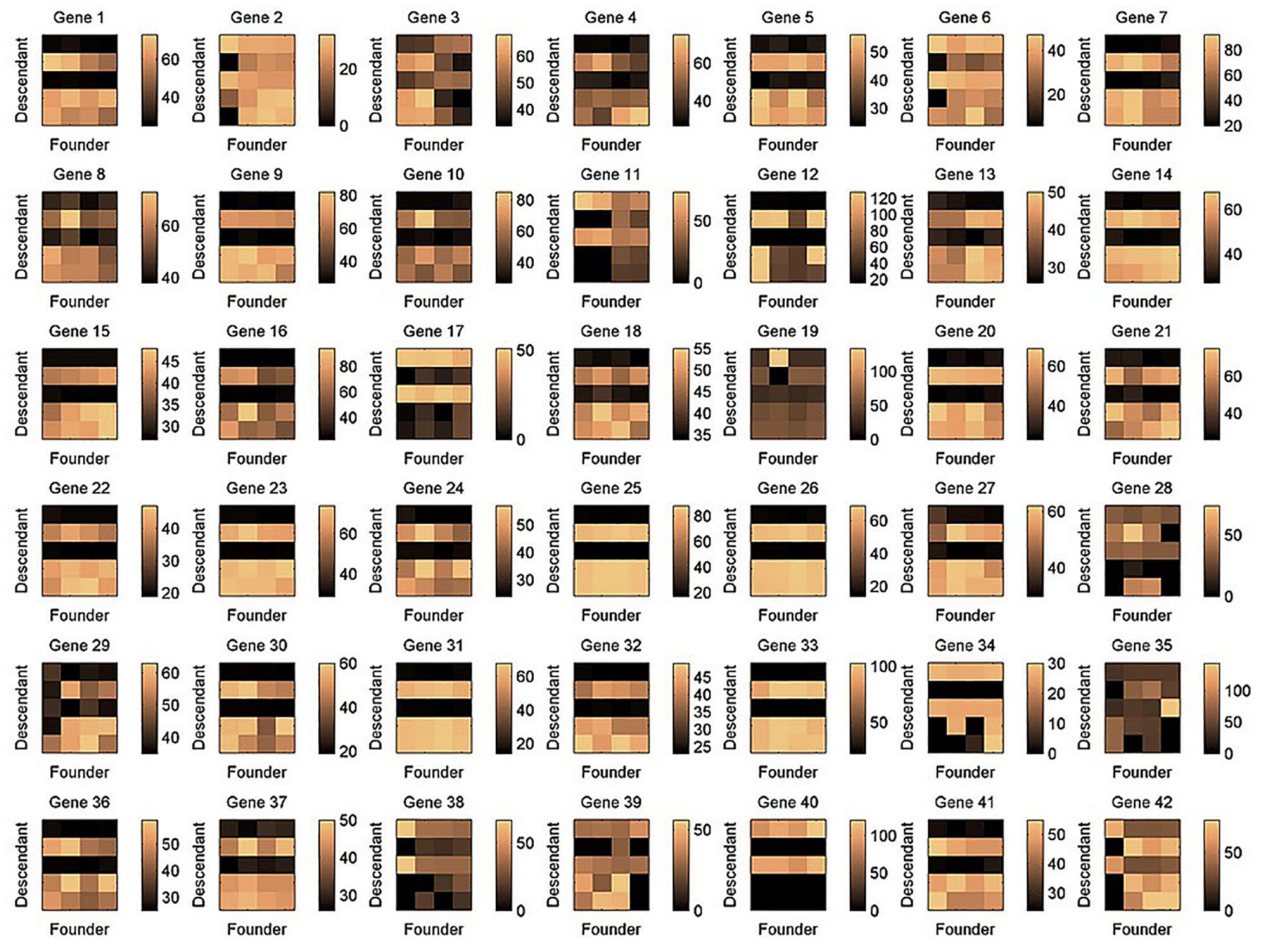
Heat maps. Heat maps of the descendant×founder matrices for the 42 genes involved in the significant co-cluster *CL*_122_ (identified in the gene×descendant×founder tensor).

A significant *δ*-*CL* (*CL*_122_), including 42 genes (**E**^1^), 5 terminal cells (**D**^2^), and 4 founder cells (**F**^2^), was detected with *δ* = 0.0702. The descendant×founder matrices for the corresponding 42 genes are displayed in Fig 12, as a series of heat maps. Likewise, similar patterns (two horizontal black lines) can be identified in the majority of these maps, defining a specific additive or multiplicative co-cluster type. The biological functions of these 42 annotated genes were further explored, using the ***GO*** terms and ***KEGG*** pathways [33]. Results are listed in Table 4, where the four annotated functional categories have long been emphasized in earlier studies of lineage tracing of C. elegans [34, 46, 49]. These functional categories may be importantly correlated to the descendant cells of ABar and ABpr branches (**F**^2^).

**Table 4:** Annotated functional categories revealed by the significant co-cluster *CL*_122_ (identified in the gene×descendant×founder tensor).

Annotated functional categories	Term	*P*-value	Benjamini adjusted *p*-value
GO:0009792	Embryonic development ending in birth or egg hatching	8.0E-4	1.1E-1
GO:0006260	DNA replication	4.1E-3	2.6E-1
GO:0006259	DNA metabolic process	4.4E-2	8.9E-1
KEGG PATHWAY	Mismatch repair	2.3E-2	3.2E-1
KEGG PATHWAY	DNA replication	4.4E-2	3.0E-1

Another significant *δ*-*CL* (*CL*_321_) with *δ* = 0.0853 include 379 genes (**E**^3^), 5 terminal cells (**D**^2^), and 4 founder cells (**F**^1^). Cell-fate changes have been well demonstrated with perturbed genes such as mex-5, gsk-3, skr-2 and cdc-25.1 [34], and now they are detected in *CL*_321_ as well. As a great number (379) of genes were involved, multiple functional categories were annotated with smaller *p*-values. The top sub-terms in ***GO*** pathway are *embryonic development ending in birth or egg hatching* (GO: 0009792), *post-embryonic development* (GO:0009791) and *nematode larval development* (GO: 0002119), each with a *p*-value less than 7.0E-30. The top three sub-terms in ***KEGG*** pathway are *spliceosome*, *RNA degradation* and *Wnt signaling*, as revealed by *CL*_321_. Notably, it successfully captures the well-acknowledged module—*Wnt signaling pathway*.

### Experiment Comparisons with Other Methods Using 2D Synthetic Data and 2D Yeast Gene Expression Data

In order to test the performance and robustness of ***HDSVS***, we implemented a series of comparion experiments. Matrices of 2D tensor data were used because most existing methods can process 2D data only and cannot be generalized to higher-order tensors easily, and multiple state-of-the-art biclustering methods were adopted for such comparisons. These methods include ***ISA*** [19], ***CC*** [2], ***FABIA*** [21], ***BSGP*** [18], ***SMR*** [29] and ***BiMax*** [20]. Specifically in our comparison experiments, **MTBA** (*MATLAB Toolbox for Biclustering Analysis*) was used as an algorithm-suite.

First, 2D synthetic data were generated based on principles in [47], resulting in matrices (500×200) with single-type biclusters (50×50). The background values are generated by a normal distribution **N**(0, 1). Comprehensively, constant, constant-row/column, additive and mulitplicative biclusters were considered and generated as follows:

1. constant biclusters, i.e. {*a*_*ij*_ = 2 ∣ *i* ∈ **I**, *j* ∈ **J**};
2. constant-row or constant-column biclusters, i.e. $\left\{a_{iJ}^{T}=\mu_{i}1_{J}|i\in I\right\}$ or {**a**_**I***j*_ = *μ*_*j*_**1**_**I**_ ∣ *j* ∈ **J**} where $1_{J}^{T}$ or **1**_**I**_ is a column vector of ones, *μ*_*i*_ and *μ*_*j*_ are drawn from a normal distribution **N**(0, 1);
3. additive-row or additive-column biclusters, i.e. $\left\{a_{iJ}^{T}=\mu_{i}1_{J}+a_{(1)J}^{T}|i,k\in I\right\}$ or $\left\{a_{Ij}=\mu_{j}1_{I}+a_{i(1)}^{T}|j,k\in J\right\}$ where **a**_(1)**J**_ (**a**_**i**(1)_) is the first row (column) of the biclusters, *μ*_*i*_, *μ*_*j*_ and each value of **a**_(1)**J**_ (**a**_**i**(1)_) are drawn from a normal distribution **N**(0, 1);
4. multiplicative-row or multiplicative-column biclusters, i.e. $\left\{a_{iJ}^{T}=\mu_{i}a_{(1)J}^{T}|i,k\in I\right\}$ or {**a**_**I***j*_ = *μ*_*j*_**a**_**I**(1)_ ∣ *j*, *k* ∈ **J**} where **a**_(1)**J**_ (**a**_**i**(1)_) is the first row (column) of the biclusters, *μ*_*i*_, *μ*_*j*_ and each value of **a**_(1)**J**_ (**a**_**i**(1)_) are drawn from a normal distribution **N**(0, 1).

**Fig 13:**
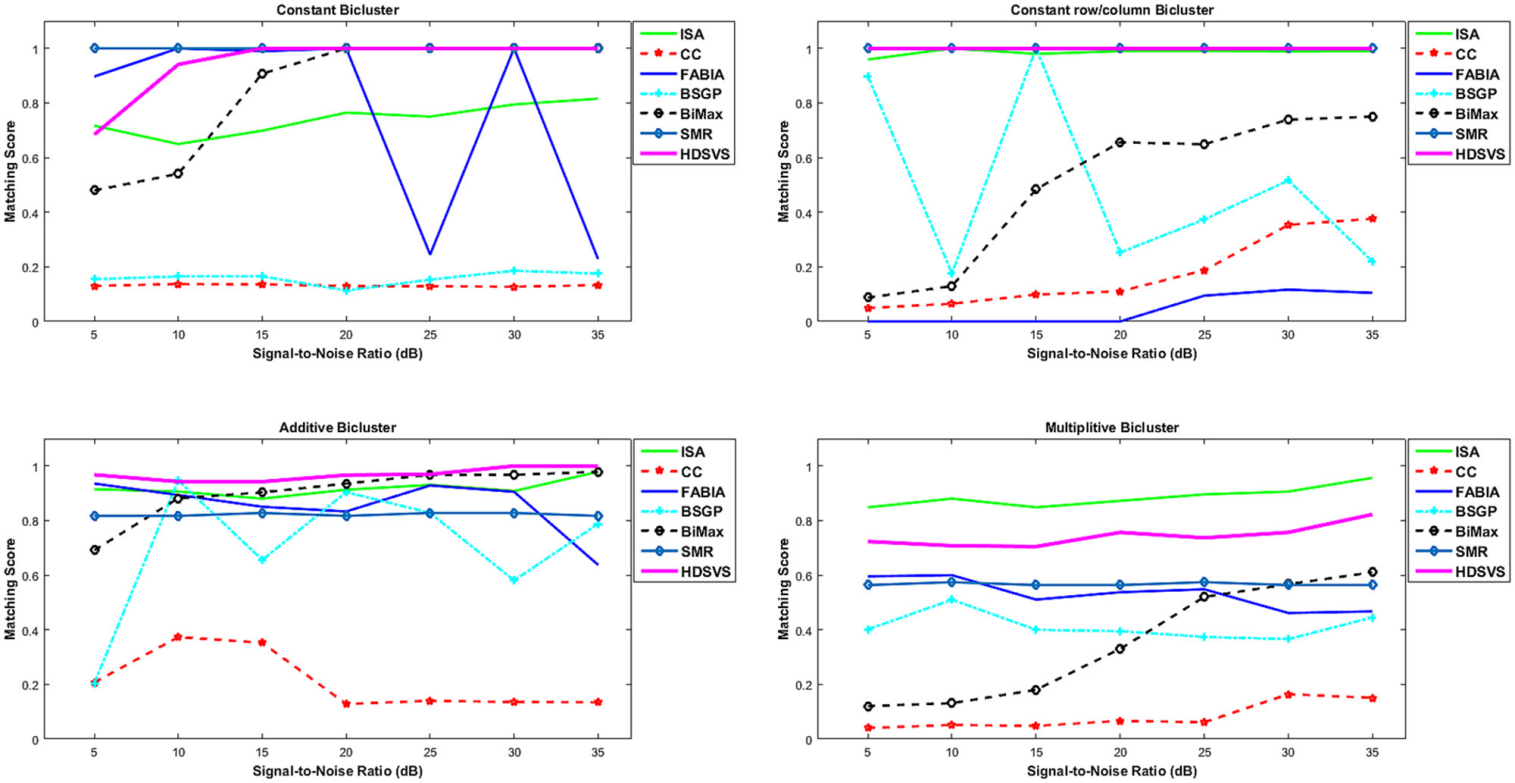
Robustness test and performance comparison for *HDSVS* and a series of other biclustering methods (*ISA*, *CC*, *FABIA*, *BSGP*, *SMR* and *BiMax*). (a) Signal-to-noise ratios (SNRs) vs. matching scores (MSs) for different biclustering methods, to search for constant biclusters. (b) SNR-MS curves for different biclustering methods, to search for constant-row/column biclusters. (c) SNR-MS curves for searching for additive biclusters. (d) SNR-MS curves for searching for multiplicative biclusters.

For each scenario involving a specific bicluster type, the matching scores of the detected biclusters and the true ones under Gaussian white noise with different SNRs (dB) were derived for each method. These SNR-MS curves of the compared methods and our algorithm are displayed in Fig 13, where parts a, b, c and d show the case concerning constant, constant-row/column, additive and multiplicative biclusters, respectively. Depending on such SNR-MS curves, the robustness of each method can be reasonably measured. As shown in Fig 13, ***HDSVS*** performs well and stably under different SNRs. Espetially, it outperforms others when additive biclusters are involved. This validates the robustness and generalization capability of our method.

**Fig 14:**
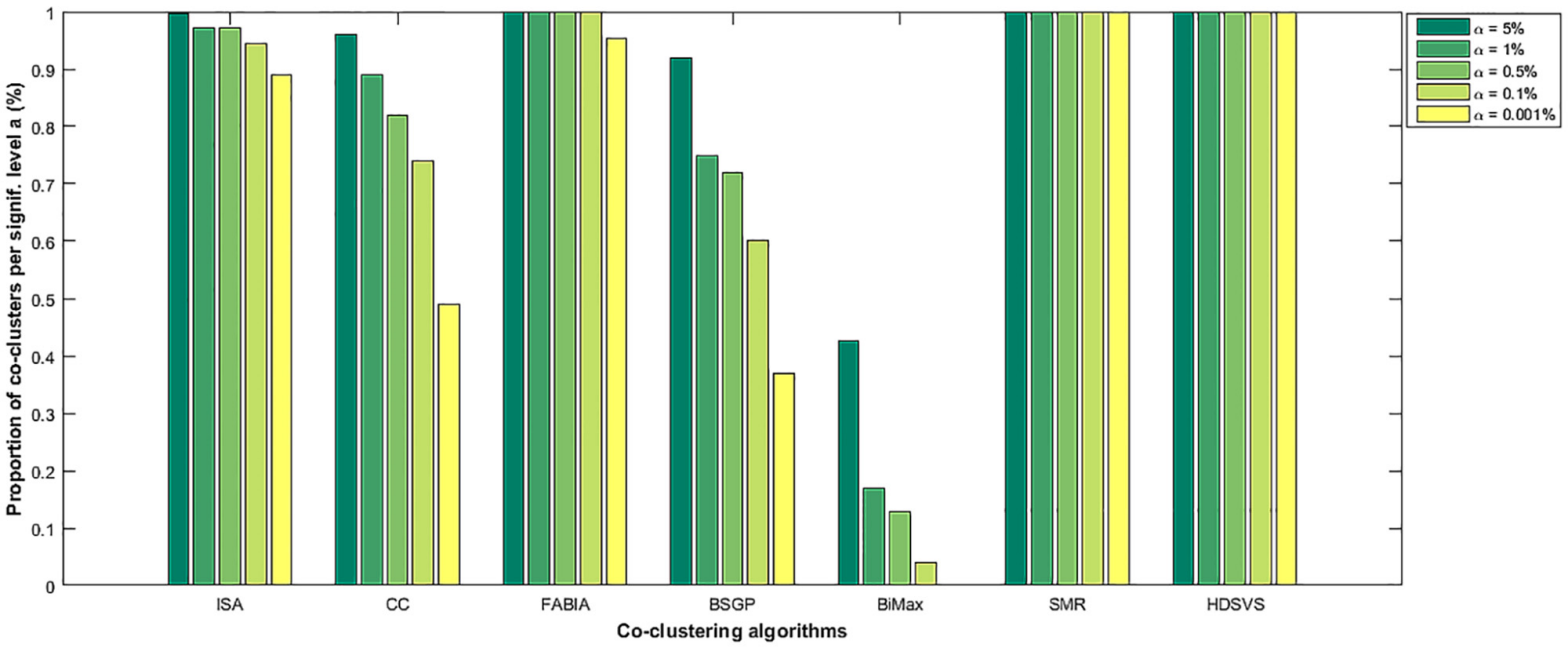
GO enrichment significance tests. Significance test results (under different thresholds) for ***HDSVS*** and existing biclustering methods (*ISA*, *CC*, *FABIA*, *BSGP*, *SMR* and *BiMax*), based on a 2D yeast gene expression tensor.

To further evaluate the statistical significance of the results generated by different methods, a biological 2D tensor, namely gene expression data of yeast cells towards different stress conditions, was employed in our comparison experiments [20]. The original microarray data (http://www.tik.ee.ethz.ch/sop/bimax/) contains 2993 genes and 173 stress conditions, and have been normalized using mean centering. A set of Perl modules for accessing GO information and evaluationg the collective annotation of a gene group to GO terms was developed in [32], based on which the statistical significance of each annotation can be calculated. Using such modules, we carried out the GO enrichment significance test for our method and each of the comparison methods. The results from different thresholds are presented in Fig 14, where ***HDSVS*** and ***SMR*** outperforms other methods in this significance test. However, ***HDSVS*** (59 seconds) is about 12 times faster than ***SMR*** (786 seconds, when *K* = 2).

## Conclusion

In this paper, we proposed a co-clustering method based on hyperplane detection in singular vector spaces (***HDSVS***), to identify co-clusters in high-order tensors. Based on linear structures of co-cluster patterns, this algorithm successfully extracted significant co-clusters (*δ*-*CL*s). Specifically, linear patterns embedded in the singular vector matrix along each mode, produced by a truncated HOSVD, were the key to co-cluster identification. These linear structures revealed by LGA showed a favorable performance in capturing the significant patterns. ***HDSVS*** was validated by multiple synthetic and biological tensors.

It is worth noting that, the performance of ***HDSVS*** was investigated with respect to different noise levels and overlapped degrees in tensors. Our method showed a robust performance on noisy tensors, due to the selection of singular vectors by the truncated HOSVD. Meanwhile, the applications of ***HDSVS*** to two biological tensors, namely the gene×patient×time tensor and the gene×descendant×founder tensor, validated its reliability in dealing with real-world applications. Especially, the genes in the detected co-clusters were significantly enriched in biologically-verified pathways and ***GO*** terms. In addition, comparisons between ***HDSVS*** and other popular methods on 2D synthetic data and 2D yeast gene expression data further showed the robustness and stability of ***HDSVS***. The experiment results show that ***HDSVS*** is an efficient method for co-cluster identification in high-order tensors. In this paper, we have used HOSVD for tensor decomposition. We can also consider the use of several other decomposition methods. For example, the dominant multidimensional subspace in tensor data can be found using higher order orthogonal iteration of tensors (HOOI) [50]. As discussed above, PARAFAC with sparse latent factors [29] has good performance for detecting co-clusters of rank 1 with low SNR. These decomposition methods can be explored in the future to improve the performance of HDSVS.

## Supporting Information

S1 Dataset Yeast gene expression data, C. elegans cell cycle data and sclerosis patients gene expression data. (MAT)
